# Distribution and abundance of the land snail *Pollicaria elephas* (Gastropoda: Pupinidae) in limestone habitats in Perak, Malaysia

**DOI:** 10.7717/peerj.11886

**Published:** 2021-07-28

**Authors:** Thor-Seng Liew, Chee-Chean Phung, Mohamad Afandi Mat Said, Pui Kiat Hoo

**Affiliations:** 1 Institute for Tropical Biology and Conservation, Universiti Malaysia Sabah, Kota Kinabalu, Malaysia; 2 Associated Pan Malaysia Cement, Chemor, Perak, Malaysia; 3 Faculty of Science, University of Malaya, Kuala Lumpur, Malaysia

**Keywords:** Elephant pupinid snails, Karst, Indochina, Malay Peninsula, Perak, Kinta Valley, Ecology

## Abstract

This study aimed to reveal the habitat variables that determine the distribution and abundance of the land snail *Pollicaria elephas* in limestone habitats in Perak, Malaysia. Seventeen plots were selected on a limestone hill to determine the effect of environmental variables on the abundance of this land snail. The environmental variables we considered included habitat (canopy cover and leaf litter thickness), topography (elevation, aspect, ruggedness, and slope), microclimate (soil temperature, air temperature, and humidity), and vegetation (abundance of respective vascular plant species). The correlation analyses suggested that the snails’ abundance was positively correlated with the abundance of the four vascular plant species: *Diospyros toposia* var. *toposoides*, *Croton cascarilloides*, *Kibatalia laurifolia*, and *Mallotus peltatus*. Plots with lower soil temperatures had more snails than plots with higher soil temperatures. Our results show that plots in the southern part of the limestone hill, in which *P*.* elephas* were absent, were similar in habitat, topography, microclimate, and vegetation to the plots in the northern part of the limestone hill, where specimens were mostly present. The absence of this species in suitable habitats may be due to their low dispersal ability rather than adverse environmental conditions.

## Introduction

There are approximately 1,000 recognised land snail species in Malaysia (http://malaypeninsularsnail.myspecies.info/, http://opisthostoma.myspecies.info/, http://borneanlandsnails.myspecies.info/). However, the ecology of the land snail species is often poorly known. To date, only a handful species from the genera *Plectostoma*, *Georissa*, *Gyliotrachela*, *Diplommatina* have been studied in terms of their growth (Berry, 1962; Berry, 1963; Liew et al., 2014b), reproduction (Berry, 1965), and demography (Berry, 1966; Schilthuizen et al., 2003).

**Figure 1:**
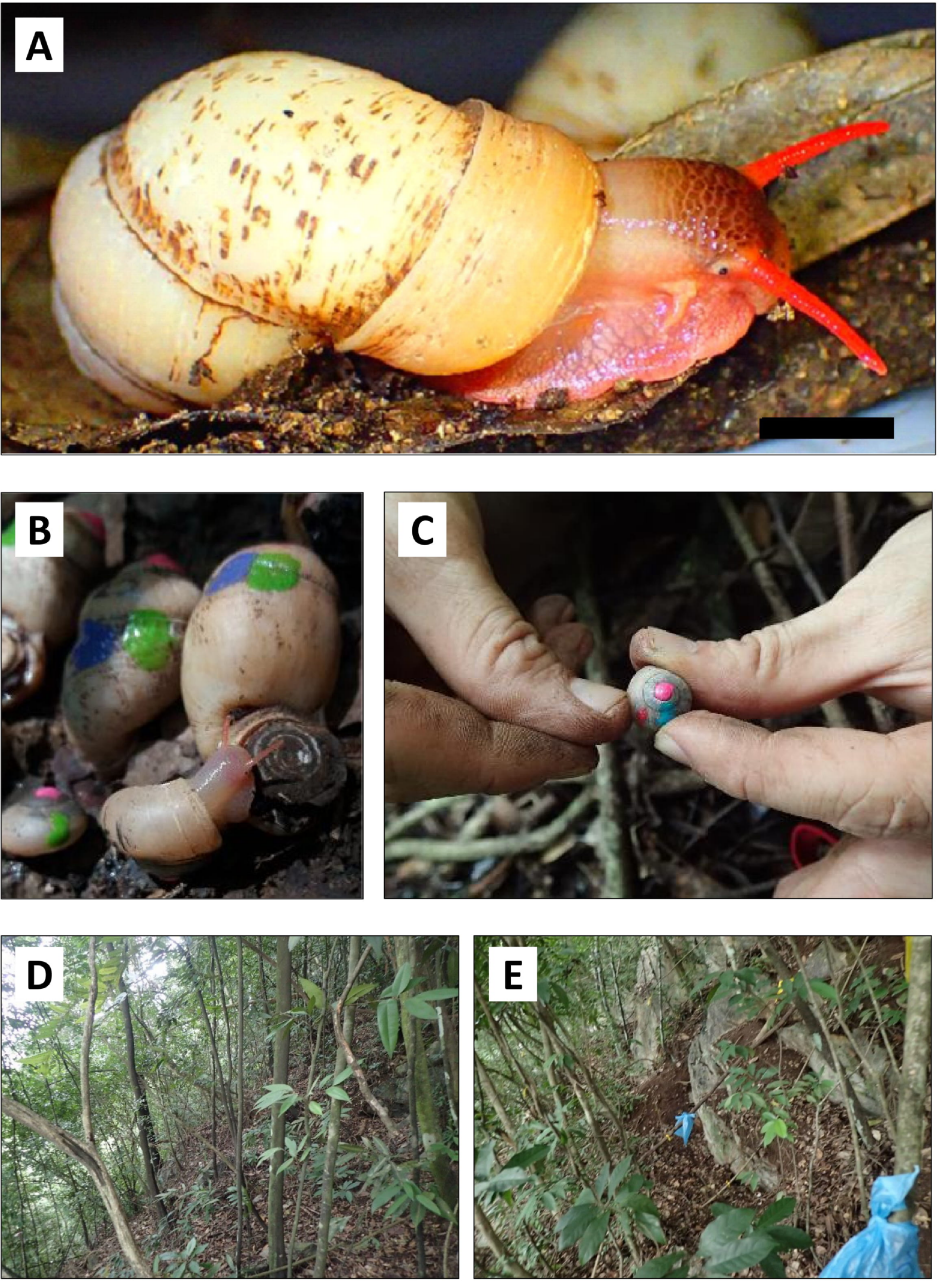
Photographs of specimens of *Pollicaria elephas* and the study site. (A) Male individual of *Pollicaria elephas*. Scale: 10 mm. (B & C) Capture-mark-recapture experiment in the field. Different colours of nail polish were used to mark the shells of living *Pollicaria elephas* that were collected during different sampling sessions. (D & E) Habitat of *Pollicaria elephas* at two localities at northern part of the limestone hill.

Land snails of the genus *Pollicaria,* commonly known as elephant pupinid snails, belong to the family Pupinidae. All seven *Pollicaria* species and subspecies from Indochina and Peninsular Malaysia are endemic to this region. *P*. *elephas* is the only *Pollicaria* species found on Peninsular Malaysia (Kongim et al., 2013) (Fig. 1A). This species was described by De Morgan (1885) in the state of Perak, Malaysia. *P*. *elephas* specimens were recorded in various localities from the limestone hills in Perak and from two other locations in Pahang (Chan, 1998; Kongim et al., 2013; Foon, Clements & Liew, 2017; Minton, Harris & North, 2017; Global Biodiversity Information Facility, GBIF, 2018).

Some aspects of *P*. *elephas*’ morphology, taxonomy, karyotypes, and geographical distribution have been studied (Pain, 1974; Chan, 1997; Kongim et al., 2010; Kongim et al., 2013). However, the ecology and small-scale distribution of this ground-dwelling species remain unknown. We found localities with very high densities of *P*. *elephas* during a preliminary survey of a limestone hill in Perak, while just tens of meters away, no snails were found. This patchy distribution is not unusual. A previous study on another similarly-sized land snail, *Limicolaria martensiana*, also showed an uneven distribution with more than 100 individuals per m^2^ at one locality at Uganda (Owen, 1969).

Studies of other macro snails from other regions show that a higher land snail abundance can be explained by vegetation or habitat characteristics, such as a denser and heterogeneous canopy and understory, higher litter humidity and thickness, older and bigger trees, rotten logs, and calcium availability (Boag, 1985; Martin & Sommer, 2004; Müller, Strätz & Hothorn, 2005; Horsák et al., 2007; Dvořáková & Horsák, 2012). The flora composition can be difficult to measure directly but may be a very useful predictor for snail communities (Dvořáková & Horsák, 2012).

We examined specific environmental parameters that may be responsible for the unevent distribution of *P*. *elephas* on a limestone hill. To date, the limestone hill in Perak is the only location where a sizeable living population of *P. elephas* is found based on several systematic samplings of land snails throughout limestone hills in Peninsular Malaysia (Chan, 1997; Clements et al., 2008; Foon, Clements & Liew, 2017). We first assessed the population size and density of *P*. *elephas* at different localities on the hill in Perak. We then examined the vegetation and topographic and microclimatic variables for each locality to characterise species-specific requirements.

## Materials and Methods

### Study site

**Figure 2:**
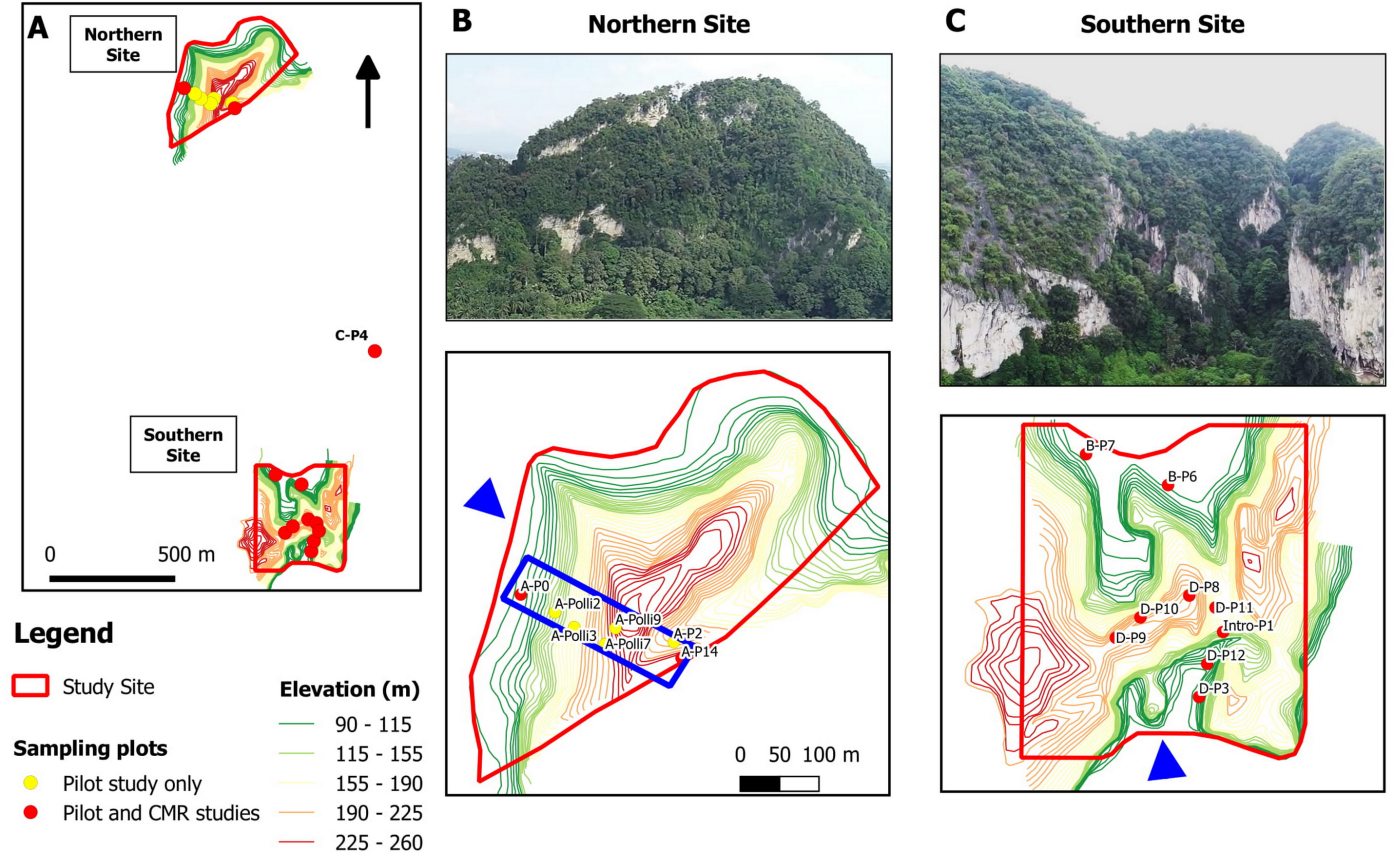
Study sites and location of the 17 studied plots at a limestone hill in the state of Perak, Malaysia. (A) Topographic map showing the seven plots at the northern part, nine plots at the southern part and one plot (C-P4) at the central part of the hill. (B) Aerial photograph from the north-western flank (blue arrow) of the hill and location of the seven plots on a topographic map. Five of the plots were selected for the capture-mark-recapture study. The blue box indicates an area of 500 m × 150 m. (C) Aerial photograph from the southern flank (blue arrow) of the hill and the location of the nine plots on a topographic map.

The study site was located on a limestone hill in Perak, Malaysia. We established a total of 17 plots, each measuring 2 m × 4 m. Seven plots were located in the northern part, nine at the southern part, and one at the central part of the hill (Fig. 2). Each plot was located next to limestone rock outcrops. A pilot survey was conducted to ensure that these plots covered habitats with different environmental variables and to identify plots with living *P. elephas* suitable for the population density study. In each plot, leaf litter was searched manually by two people for over 20 min to find living snails and empty shells. Environmental variables for each of the 17 plots were measured during the pilot survey on 11 May 2018.

### Spatial distribution and population density

We did not find any living *P. elephas* or empty shells in the 11 of the 17 examined plots during the pilot survey (Tables S1). We used the capture-mark-recapture method (CMR) to study the population of *P*. *elephas* in five of the six plots with living snails. One of the plots (AP-0) was inaccessible after the pilot survey so the CMR study could not be conducted in that plot. The captured *P. elephas* were marked with different colours of nail polish (Figs. 1B and 1C) during different sampling sessions. The snails were then released back to their respective plots, and plots were resampled after 10 to 15 days. The CMR was conducted four times for all plots with the exception of one plot (AP-2), where only three CMR sessions took place. The first sampling was conducted on 9 July 2018 and the first recapture was on 19 July 2018, the second recapture occurred on 1 August 2018, and the third recapture took place on 16 August 2018. The collected snails from the following demographics were examined: juvenile (<3 whorls), subadult (3–5 whorls), and adult (with aperture lip). All living snails caught were released back to their respective plots while the empty shells were collected and deposited in the BORNEENSIS collection of Universiti Malaysia Sabah.

We calculated the *P. elephas* population density by counting the living snails in individual plots during the different CMR sessions. The population size of *P*. *elephas* was estimated based on the data collected using the Schnabel index:

• N = total number of snails (unknown)
• C = number of snails captured on the first sampling
• M = number of snails captured on subsequent sampling
• R = number of snails captured on both samplings

Multiple marks and recaptures ensured a more accurate estimation of the population size. The Schnabel method (Alcoy, 2013), which allows multiple capture-recapture encounters, was used:

\begin{eqnarray*}N& = \frac{\sum _{i=1}^{m}{M}_{i}{C}_{i}}{\sum _{i=1}^{m}{R}_{i}} \end{eqnarray*}

\begin{eqnarray*}{M}_{i}& =\text{Total number of previously marked snails at time}~i \end{eqnarray*}

\begin{eqnarray*}{C}_{i}& =\text{The number caught at time}~i \end{eqnarray*}

\begin{eqnarray*}{R}_{i}& =\text{The number of marked snails caught at time}~i \end{eqnarray*}

### Environmental variables

We studied four main environmental variables, namely habitat, topography, microclimate, and vegetation. All of the variables were measured for all 17 plots, with the exception of the microclimate. We measured leaf litter thickness by averaging litter thickness at eight points within each plot for habitat variables and estimated the percentage of canopy cover. To obtain topographic variables, we created a digital elevation model (DEM) of 4 m^2^ cell-size based on a 5-meter interval contour map for each of the northern and the southern study site using Triangular interpolation (TIN) in QGIS (ver. 2.18.24; QGIS Development Team, 2018). We then used terrain analysis tools to derive topographic features including slope, aspect, and the terrain ruggedness index (a quantitative measurement of terrain heterogeneity) (Riley, DeGloria & Elliot, 1999). Topographic parameters were extracted for each sampling plot using the ‘Add raster values to point’ setting in SAGA (Conrad et al., 2015).

### Microclimatic variables

We installed climatic HOBO data loggers to record air temperature and humidity (HOBO MX2301 Temperature/RH) approximately one meter above the ground for eight of the 17 plots (A-Polli3, A-Polli9, D-P1, D-P3, D-P8, D-P9, D-P11, D-P12). We recorded the soil temperature using HOBO MX2303 temperature sensors for the same eight plots. External sensors were fully covered by leaf litter. These eight plots have been chosen, as they represent localities with both absence and presence data and different population densities of these snails. Living specimens were found in two northern plots, namely, plot A-Polli9, which had a higher number of snails (15–16 individuals) and plot A-Polli3, which had a lower number of snails (1–2 individuals). There were no living specimens found in the five southern plots. The climatic parameters were logged every 10 min in July 2018. Data from the two soil temperature loggers could not be retrieved due to damage by rain and wildlife.

### Vegetation data

We counted and identified all the vascular plants with diameter at breast height (DBH) above one cm within a 5-meter radius from the centre of each of the 17 plots to obtain the number of vascular plant individuals and the number of vascular plant species of each plot. Voucher specimens were collected for each species and were subsequently identified by P.K. Hoo based on the reference materials at the Herbarium of Forest Research Institute Malaysia (FRIM).

### Data analysis

Principal component analysis (PCA) was conducted to assess the degree of habitat heterogeneity among the 17 plots based on the two habitat variables (leaf litter thickness, and canopy cover), four topographic variables (elevation, slope, aspect, and ruggedness index) and two vegetation variables (number of vascular plant individuals and number of vascular plant species). The abundance of vascular plant species was not included in the PCA analysis due to the number of missing values in the dataset and the absence of certain plant species in plots. We visually explored the PCA plot for habitat heterogeneity according to the plots’ locations on the limestone hill (northern part, southern part, and central part). The analysis was done using R (File S2).

Correction tests were performed to examine any significant relationships between the abundance of snails and each of the habitat, vegetation, and topographic variables. We excluded 43 vascular plant species that were recorded only in one plot before statistical analysis to obtain vegetation data. The final dataset consisted of the abundance data for 20 vascular plant species from 17 plots.

As the data were not normally distributed, we used Spearman correlation testing based on both null-hypothesis significance testing and corroborated our analysis by using the Bayes factor (BF10) (Kass & Raftery, 1995). Our conclusion is based on the inference of both frequentist (*p* values) and Bayesian (Bayes factor) analyses. All analyses were performed using JASP software version 0.12.2 (JASP Team, 2020; File S3).

There was either complete or partial missing microclimatic data from July for some of the plots, so we did not calculate the mean values for each of the microclimatic variables per month. Hence, we could not perform rigorous statistical analysis to test the relationship between the microclimatic variables and the abundance of snails in the plot. Nevertheless, we explored the relationships between the abundance of snails and microclimatic variables by plotting the mean of each sampled plot’s daily microclimatic variables patterns. We calculated the daily mean air humidity, minimum air humidity, mean air temperature, maximum air temperature, mean soil temperature, and maximum soil temperature.

## Results

### Spatial distribution and population density of the snails

The numbers of living specimens collected over the four CMR sessions are shown in File S1). Living specimens were found in six out of seven plots on the northern part of the hill; none were found in plots on the central and southern parts (File S1). The smallest marked specimen was nine mm (shell width), and the majority of the marked snails were subadult and adult (File S1). The recapture rates were greater than 80% for the three plots with more than ten snails recorded during the pilot survey and the first capture session of CMR (A-Polli2, A-Polli7, and A-Polli9, see Tables S1–S4 in, except for two recapture sessions in plot A-Polli2 (23% and 67%). The recapture rates were between 50% and 100% for the two plots with less than ten snails.

Of the five plots examined in the CMR study, plot A-Polli2 had the highest population size of *Pollicaria elephas* (Table 1), and the calculated population density was estimated to be approximately 57 individuals for that plot and its surrounding area. The highest number of snails recorded per sampling event per plot was 26 specimens in plot A-Polli2 (Table S2). The snails’ population density in the sampling plots varied only slightly during the different sampling sessions for each plot (File S1).

**Table 1:** Population estimation for *Pollicaria elephas* for each plot based on capture-markrecapture technique and Schnabel index.

Plots	Population density^a^	Population estimation by CMR (number of snails)
A-Polli2	23.0 ± 3.4	56.9
A-Polli9	15.8 ± 1.6	20.8
A-Polli7	17.4 ± 2.8	19.5
A-Polli3	1.4 ± 0.5	2.0
A-P2	6.3 ± 2.1	12.1

**Notes.**

a Average number of snails ± standard deviation for all of the CMR sessions at each 8 m^2^ plot.

### Effect of environmental variables on snail occurrence and abundance

**Figure 3:**
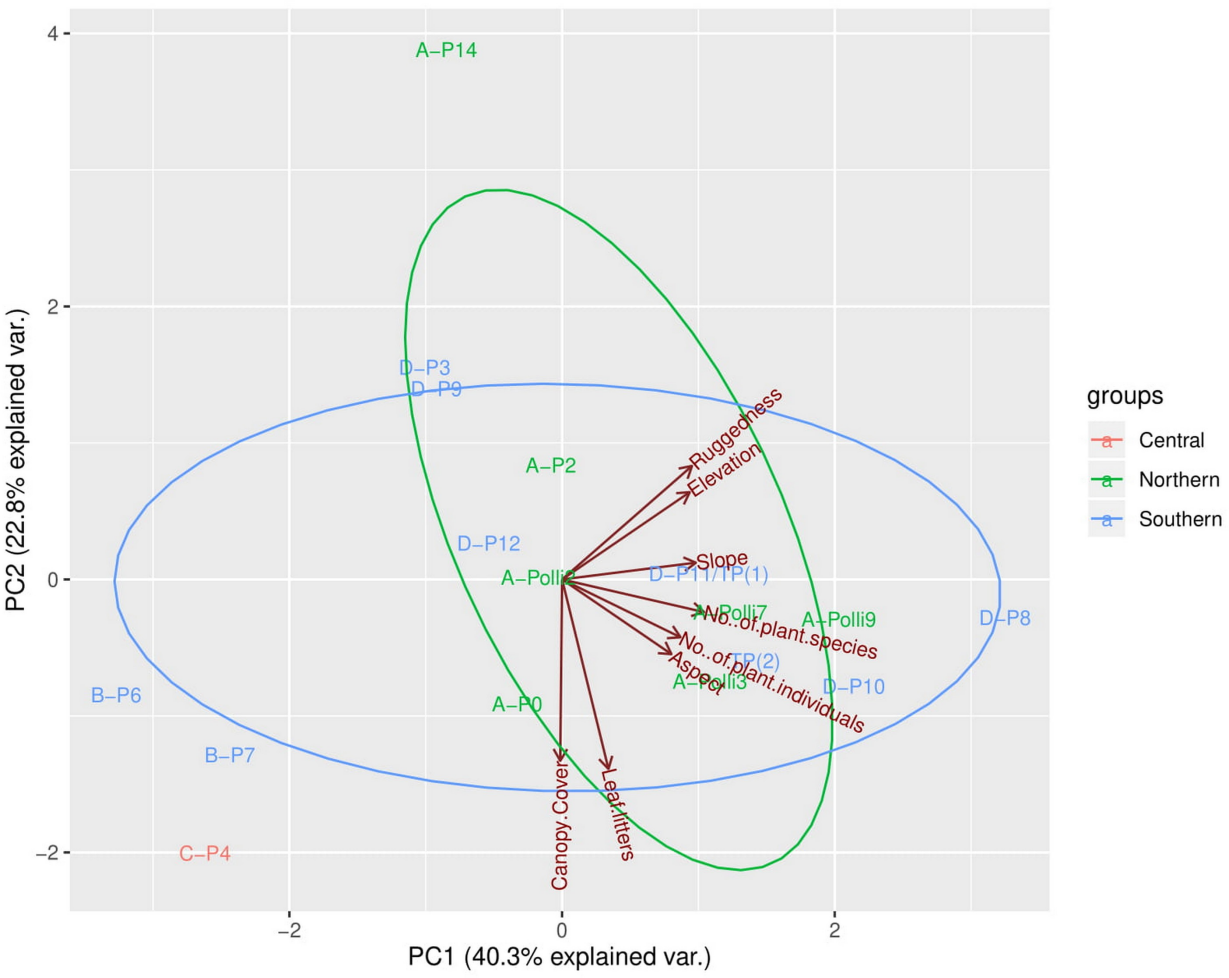
Principal components analysis (PCA) plot of the first two axes for habitat, topography, and vegetation variables for the 17 plots. The colour of the labels represents the plot location on the limestone hill and red vectors represent the habitat, topography, and vegetation variables. No individuals of *Pollicaria elephas* were found in plots at the southern and central part of the limestone hill, while at the northern part living snails were found in six of the seven plots (none were found in A-P14).

The first three PCA axes explained 78.7% of the habitat, topography, and vegetation variations between plots (Fig. 3, Files. S4, S5). As shown in Fig. 3, the PCA plot did not show apparent differences between the plots in the northern part (most of the plots with living *P*. *elephas)* and the southern part (all plots without living *P*. *elephas)* on the limestone hill. The abundance of *P. elephas* per plot was not correlated with canopy cover, leaf litter thickness, elevation, aspect, slope, and the ruggedness of the habitat (Table 2).

**Figure 4:**
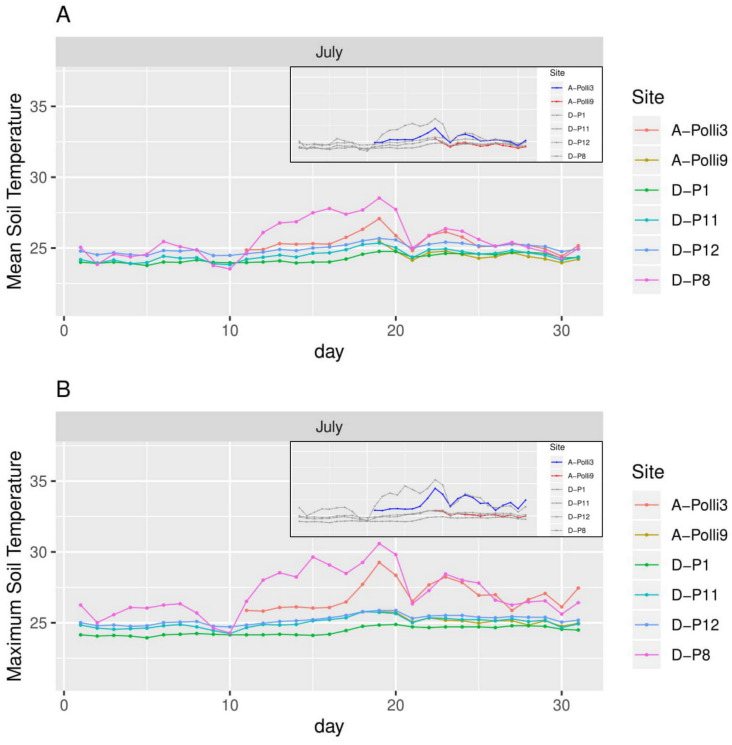
Soil temperature for each day in July 2018 in the six plots. A-Polli3 and A-Polli9 are the plots with living *Pollicaria elephas* land snails, while the other four plots are without living *P*. *elephas* land snails. The insets represent the same plots with a different colour legend for the plots with living snails. (A) Mean soil temperature for each day. (B) Maximum soil temperature for each day.

The plot A-Polli9 with the higher number of living snails (15–16 individuals) had a soil mean temperature lower than 25 °C, and maximum temperature lower than 26 °C (Fig. 4). However, the two plots, namely D-P1 and D-P11, at the southern part of the hill with no snails recorded had a similar mean and maximum soil temperature (∼25 °C) to the plot A-Polli9.

**Table 2:** Correlation between the abundance of *Pollicaria elephas* for each plot with the habitat and topographic parameters.

**Habitat and topographic variables**	**Bayesian Kendall’s Tau Correlations**	**BF_1__0_**	**Kendall’s Tau Correlations**	*p*-value
Number of vascular plant individuals for each plot	−0.186	0.512	−0.186	0.353
Number of vascular plant species for each plot	0.128	0.392	0.128	0.525
Canopy cover (%)	0.261	0.84	0.261	0.216
Leaf litter thickness (cm)	0.154	0.437	0.154	0.436
Elevation (meters)	0.183	0.504	0.183	0.355
Aspect (counter-clockwise in degrees from 0 (due north) to 360 (again due north))	0.086	0.343	0.086	0.662
Ruggedness index	0.092	0.372	0.092	0.669
Slope (degrees)	0.241	0.721	0.241	0.224

**Figure 5:**
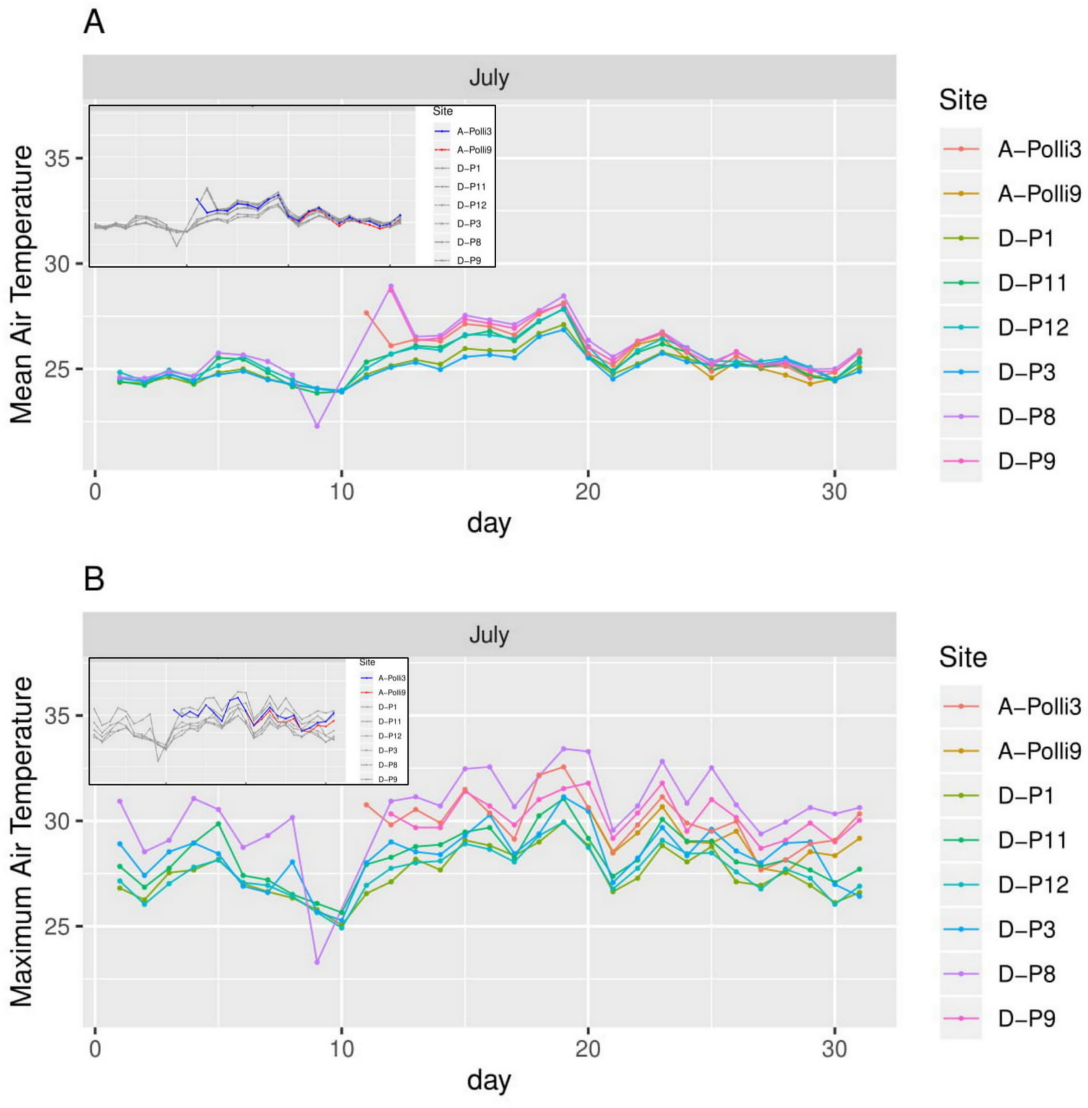
Air temperature for each day in July 2018 in the eight plots. A-Polli3 and A-Polli9 are the plots with living *Pollicaria elephas* land snails, while the other six plots are without living *P. elephas* land snails. The insets represent the same plots with a different colour legend for the plots with living snails. (A) Mean air temperature for each day. (B) Maximum air temperature for each day.

**Figure 6:**
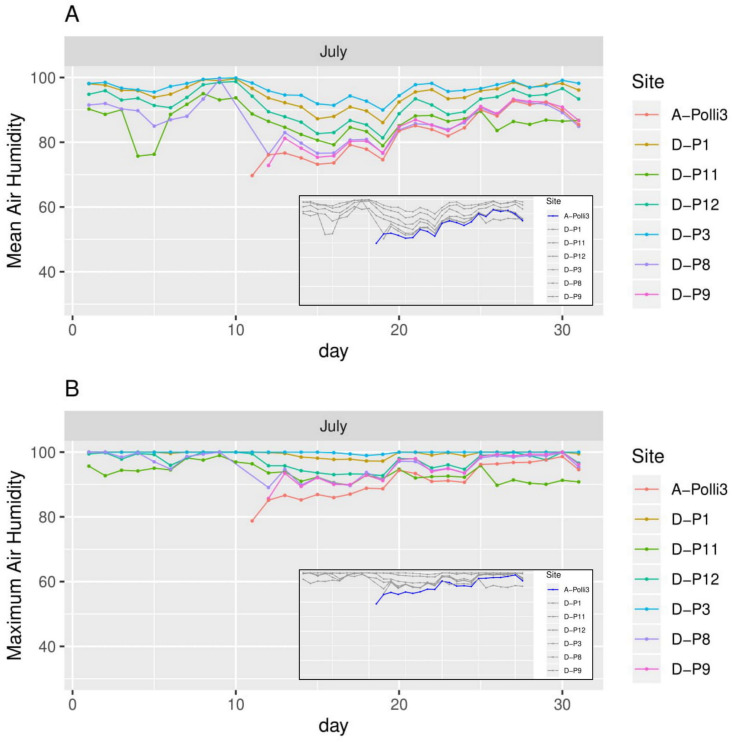
Humidity for each day in July 2018 in the seven plots. Plot A-Polli3 is the plot with living *Pollicaria elephas* land snails, while the other six plots are without living *P. elephas* land snails. The insets represent the same plots with a different colour legend for the plots with living snails. (A) Mean humidity for each day. (B) Maximum humidity for each day.

All the plots at the southern and northern parts of the hill had a similar mean temperature with differences smaller than 1 °C during most of the days, with the exception of plots D-P1 and D-P3 (Fig. 5). There were no significant differences in the mean temperature among plot A-Polli9 with a high number of living snails (15–16 individuals), plot A-Polli3 with few living snails (one to two individuals), and other plots without living snails. The plots with a higher mean humidity (85%–100%) at the southern part of the hill did not harbour *P*. *elephas* as compared to plots with lower humidity (75%–93%) at the northern part of the hill (Fig. 6).

### Association between the abundance of *P. elephas* and vegetation

Sixty-three taxa of vascular plants were recorded in the 17 plots. Species identifications were obtained for 46 species. The identity of 14 species could only be confirmed at the genus level, and the remaining three species could not be identified. Altogether 43 vascular plant species were recorded in only one plot, of which 27 species were singletons (Table 3). The number of species per plot ranged between three and 11 species, and the number of individuals ranged between four and 42 (File S4).

The abundance of four vascular plant species were positively correlated with the abundance of the land snails based on the null-hypothesis significance testing (*p* < 0.05) and Bayes factor (BF10) (Table 4, Table S7). Of these four species, *Diospyros toposia* var. *toposoides* (Ebenaceae) was the only plant species found in plots with and without living snails. Two plant species, namely, *Croton cascarilloides* (Euphorbiaceae) and *Kibatalia laurifolia* (Apocynaceae), were recorded only in two plots (A-P0 and A-Polli9). Another plant species, *Mallotus peltatus* (Euphorbiaceae), was recorded in only three plots (A-P0, A-P2 and A-Polli2). The total number of vascular plants and plant species was not correlated with the abundance of snails in the plots (Table 2).

## Discussion

Synecology studies have focused on the association of habitat features and the composition of communities of land snails (Müller, Strätz & Hothorn, 2005). However, autecology studies on single species in their natural habitat are scarce. The two different approaches of ecology have developed independently, although the knowledge of both is necessary to understand the ecology of an individual population within a species or the whole ecosystem. A broader understanding of a species’ biogeography starts with the knowledge of the species’ autecology on a local scale (Hugall et al., 2002). Unfortunately, studies on the responses to environmental variables by individual species of large land snails in the tropical ecosystem are lacking (Horsák et al., 2007).

### Spatial distribution and population density of *P. elephas*

The capture-mark-recapture technique has been used for estimation of population size and density for land snails (Blinn, 1963; Hänsel, Walther & Plachter, 1999; Standish, Bennett & Stringer, 2002; Parkyn, Brooks & Newell, 2014). Previous studies on other land snails showed that the capture rate using the CMR technique can be very high, with up to an 85% recapture rate after one year (Kleewein, 1999). In our study, the recapture rates varied by plot. Using this technique, we found that *P*. *elephas* can achieve high population densities, between three and four individuals per square meter, in suitable habitats at the northern part of the examined limestone hill. We could not find other population density studies on similarly-sized caenogastropod land snails for comparison. However, studies on similarly-sized pulmonate land snails showed that snails can occur in very low densities: less than one individual per m^2^ to very high densities of up to 100 individuals per m^2^ for *Limicolaria martensiana* (Owen, 1969). However, Owen (1969) did not investigate environmental factors that could determine the variation of densities in different population.

We could not find *P*. *elephas* in the southern part of the limestone hill, while large populations occurred on the northern part of the limestone hill. All examined plots were on the same limestone outcrop, with generally similar climatic condition, soil conditions, and land snail communities (Foon, Clements & Liew, 2017). Furthermore, there were no apparent differences in environmental variables in the microclimate, topography, habitat, and vegetation between the northern and southern part of the limestone hill.

**Table 3:** Checklist and the number of individuals per vascular plant species in each plot.

	**Family and species**	**Plots**																	**The total number of individuals of each species in 17 plots**	**The total number of plots where this species was present**
		**Plots with living*Pollicaria elephas*snails**						**Plots without** **living*Pollicaria elephas*snails**												
		**A-P0**	**A-P2**	**A-Polli2**	**A-Polli3**	**A-Polli7**	**A-Polli9**	**A-P14**	**B-P6**	**B-P7**	**C-P4**	**D11**	**D-P10**	**D-P12**	**D-P3**	**D-P8**	**D-P9**	**Intro-P1**		
	**Anacardiaceae**																			
1	*Mangifera* sp. 1												1			1			**2**	**2**
	**Annonaceae**																			
2	Annonaceae sp. 1													1					**1**	**1**
3	*Cananga odorata*									1									**1**	**1**
4	*Orophea cuneiformis*	2																	**2**	**1**
5	*Polyalthia* sp. 1											4						2	**6**	**2**
6	*Polyalthia* sp. 2		1																**1**	**1**
7	*Xylopia* sp. 1							1								1			**2**	**2**
	**Apocynaceae**																			
8	*Kibatalia laurifolia*^a^	1					1												**2**	**2**
	**Bignoniaceae**																			
9	*Radermachera pinnata acuminata*															1			**1**	**1**
	**Celastraceae**																			
10	*Euonymus javanicus*						1												**1**	**1**
	**Clusiaceae**																			
11	*Garcinia cowa*															1			**1**	**1**
	**Cycadaceae**																			
12	*Cycas clivicola*					1													**1**	**1**
	**Dipterocarpaceae**																			
13	*Vatica kanthanensis*					1							2					1	**4**	**3**
	**Ebenaceae**																			
14	*Diospyros frutescens*											1							**1**	**1**
15	*Diospyros toposia* var. toposoides^a^	3		2	7	8	7					2		1				3	**33**	**8**
16	*Diospyros transitoria*		2																**2**	**1**
	**Euphorbiaceae**																			
17	*Croton cascarilloides*^a^	2					1												**3**	**2**
18	*Macaranga gigantea*														1				**1**	**1**
19	*Macaranga tanarius*									1	3								**4**	**2**
20	*Mallotus barbatus*							3											**3**	**1**
21	*Mallotus brevipetiolatus*								7	12		3		5				2	**29**	**5**
22	*Mallotus peltatus^a^*	6	7	2															**15**	**3**
23	*Pimelodendron griffithianum*													1					**1**	**1**
	**Lamiaceae**																			
24	*Vitex siamica*															2			**2**	**1**
	**Lauraceae**																			
25	*Dehaasia cuneata*						1									1			**2**	**2**
	**Leguminosae**																			
26	*Archidendron jiringa*					1													**1**	**1**
27	*Bauhinia* sp.										1								**1**	**1**
	**Malvaceae**																			
28	*Hibiscus macrophyllus*							8											**8**	**1**
29	*Leptonychia caudata*									6		5						5	**16**	**3**
30	*Pterospermum acerifolium*					1													**1**	**1**
31	*Sterculia rubiginosa*													1					**1**	**1**
	**Melastomataceae**																			
32	*Memecylon lilacinum*												8						**8**	**1**
	**Meliaceae**																			
33	*Aglaia grandis*					1													**1**	**1**
	**Moraceae**																			
34	*Ficus fistulosa*							1											**1**	**1**
35	*Ficus hispida*										2								**2**	**1**
36	*Ficus schwarzii*									1									**1**	**1**
37	*Ficus* sp. 1										1								**1**	**1**
38	*Ficus* sp. 2														2				**2**	**1**
	**Palmae**																			
39	*Arenga westerhoutii*		4												1				**5**	**2**
40	*Borassodendron machadonis*		1																**1**	**1**
41	*Caryota mitis*							1											**1**	**1**
	**Pandanaceae**																			
42	*Pandanus piniformis*												5			3	2		**10**	**3**
	**Phyllanthaceae**																			
43	*Bridelia tomentosa*							1					10			25	8		**44**	**4**
44	*Cleistanthus gracilis*			1	15				5				3						**24**	**4**
45	*Cleistanthus myrianthus*		1															6	**7**	**2**
	**Primulaceae**																			
46	*Ardisia* sp.											2							**2**	**1**
47	*Myrsine perakensis*															1			**1**	**1**
	**Rubiaceae**																			
48	*Aidia densifolia*			3	2								7			3	6		**21**	**5**
49	*Canthium* sp. 1															3			**3**	**1**
50	*Canthium* sp. 2																	4	**4**	**1**
51	*Saprosma* sp.						1												**1**	**1**
	**Rutaceae**																			
52	*Micromelum minutum*																	1	**1**	**1**
53	*Murraya paniculata*				1	1	1			1									**4**	**4**
54	Rutaceae sp. 1																2		**2**	**1**
55	Rutaceae sp. 2																	1	**1**	**1**
	**Salicaceae**																			
56	*Homalium dasyanthum*						2												**2**	**1**
57	*Homalium grandifllorum*												1						**1**	**1**
	**Sapindaceae**																			
58	*Paranephelium spirei*	1																	**1**	**1**
	**Sapotaceae**																			
59	*Isonandra perakensis*												2						**2**	**1**
	**Violaceae**																			
60	*Rinorea bengalensis*	2	2	3	4	3	2		5			2		7				6	**36**	**10**
	**Indet**																			
61	Indet sp. 1						2												**2**	**1**
62	Indet sp. 2													1					**1**	**1**
63	Indet sp. 3													2					**2**	**1**

**Notes.**

a Plant species were a positive correlation between the abundances of plants and snails was found.

**Table 4:** Correlation between the abundance of *Pollicaria elephas* for each plot with the abundance of the vascular plant species.

Family	Species	**Occurrence in the 17 plots**	Bayesian Kendall’s Tau Correlations	BF_10_	Kendall’s Tau Correlations	*p*-value
Anacardiaceae	*Mangifera* sp. 1	2	−0.243	0.735	−0.243	0.295
Annonaceae	*Polyalthia* sp. 1	2	−0.239	0.714	−0.239	0.296
Annonaceae	*Xylopia* sp. 1	2	−0.243	0.735	−0.243	0.295
Apocynaceae	*Kibatalia laurifolia*	2	0.527*	17.738	0.527*	0.023
Dipterocarpaceae	*Vatica kanthanensis*	3	−0.050	0.320	−0.050	0.826
Ebenaceae	Diospyros toposia var. *toposoides*	8	0.519*	15.604	0.519*	0.015
Euphorbiaceae	*Croton cascarilloides*	2	0.499*	11.617	0.499*	0.030
Euphorbiaceae	*Macaranga tanarius*	2	−0.239	0.714	−0.239	0.296
Euphorbiaceae	*Mallotus brevipetiolatus*	5	−0.398	3.139	−0.398	0.070
Euphorbiaceae	*Mallotus peltatus*	3	0.547*	23.867	0.547*	0.016
Lauraceae	*Dehaasia cuneata*	2	0.162	0.454	0.162	0.485
Malvaceae	*Leptonychia caudata*	3	−0.302	1.167	−0.302	0.187
Palmae	*Arenga westerhoutii*	2	0.060	0.325	0.060	0.794
Pandanaceae	*Pandanus piniformis*	3	−0.298	1.133	−0.298	0.187
Phyllanthaceae	*Bridelia tomentosa*	4	−0.350	1.854	−0.350	0.116
Phyllanthaceae	*Cleistanthus gracilis*	4	0.131	0.397	0.131	0.556
Phyllanthaceae	*Cleistanthus myrianthus*	2	0.020	0.310	0.020	0.931
Rubiaceae	*Aidia densifolia*	5	0.000	0.308	0.000	1.000
Rutaceae	*Murraya paniculata*	4	0.370	2.279	0.370	0.112
Violaceae	*Rinorea bengalensis*	10	0.257	0.809	0.257	0.223

**Notes.**

* BF_10_ > 10.

** BF_10_ > 30.

*** BF_10_ > 100

* *p* < 0.05

** *p* < 0.01

*** *p* < 0.001

a The variance in *Macaranga tanarius* is equal to 0.

However, it is unclear why the snails from high-density spots did not migrate to the other spots at the same hill with similar habitat. The dispersal ability of this species was not determined in this study. Nevertheless, the dispersal distances for other similar-sized land snails are very short, ranging from meters to tens of meters per year (Baur, 1986; Schilthuizen et al., 2005; Edworthy et al., 2012; Ozgo & Bogucki, 2011; Kramarenko, 2014). One possible explanation could be the homing behaviour found in certain snail species (Rollo & Wellington, 1981; Tomiyama, 1992; Stringer, Parrish & Sherley, 2018) with highly specialised habitat requirements. These species are not migrating far from their favoured spot and can have narrow-ranged endemics occurring unevenly across a large landscape. Another possible explanation may be that an unfavourable habitat prevents dispersal of this species to isolated spots with suitable habitat.

Although the environmental variables included in this study were unlikely to determine the absence and presence of this species in different parts of the hill, the heterogeneity of population densities in the plots at the northern part of the hill showed that higher abundance of *P*. *elephas* could be associated with lower soil temperatures. This is expected as *P*. *elephas* is a ground-dwelling land snail. From our observation on the snails’ behaviour in the field and in captive populations, snails were active during the night where they were seen feeding on leaf litter. In the day, the snails could be found burying themselves underneath leaf litter. Living snails in the field were never found attached to vegetation or rocks above ground. Hence, we can assume that a constant and relatively low soil ground temperature and a lower air temperature and higher humidity at night is important for the population to thrive File S8).

### Association between snail abundance and abundance of plant species

Previous studies on the association between plants and specific land snail species have been conducted outside of the tropical regions (Blinn, 1963; Pollard, 1975; Cowie, 1985; Hänsel, Walther & Plachter, 1999; Standish, Bennett & Stringer, 2002; Burrell, Scott & Yen, 2007; Horsák, Škodová & Cernohorsky, 2011; Parkyn & Newell, 2013; Parkyn, Brooks & Newell, 2014). Most of these studies suggested that plants act as shelters for land snails (Blinn, 1963; Pollard, 1975; Cowie, 1985; Standish, Bennett & Stringer, 2002; Burrell, Scott & Yen, 2007; Parkyn, Brooks & Newell, 2014).

The abundance of *P*. *elephas* is positively associated with a relatively common vascular plant, *Diospyros toposia* var. *toposoides*, on the limestone hill. The other three comparatively uncommon species, namely, *Croton cascarilloides*, *Kibatalia laurifolia*, and *Mallotus peltatus* were also positively correlated with the snails’ abundance. All of the three species are only found in plots with living snails (Table 3). However, it is important to note that due to the nature of high heterogeneity of vascular plants in the forest, as well as the 43 vascular plant species that were recorded in only one plot, there were only two plots for *Kibatalia laurifolia* and *Croton cascarilloides,* respectively, and three plots for *Mallotus peltatus*. Also, this indicates that the presence of the three plant species is not necessary for the presence of the snails as these plants were not found in other plots where snails were present. Hence, future studies of carefully-selected sites with different abundances of living snails and our identified species should be conducted to verify the possible causal relationship.

A plausible explanation for this relationship could be that the leaf litter from these plant species is suitable for the snails’ diet. However, it is also possible that rather than a direct causal relationship, both plants and snails prefer the same parameters of the local environment. A specially designed experiment is needed to test these hypotheses. To our knowledge, there were no *in situ* experiments on food preferences of land snails conducted in the field. However, there have been laboratory experiments conducted with decaying leaves of selected plant species (Puslednik, 2002; Proćków et al., 2013). *In situ* experiments on food preferences in a tropical rainforest are challenging because identifying leaf litter from plants is difficult, as plants are very species rich even within a small area, as was shown in this study (Crowther, 1982; Crowther, 1987a; Crowther, 1987b). We cannot rule out the hypothesis that plants and leaf litters provide shelter for land snails (e.g., Blinn, 1963; Pollard, 1975; Cowie, 1985; Standish, Bennett & Stringer, 2002; Burrell, Scott & Yen, 2007; Parkyn, Brooks & Newell, 2014).

There were single-species land snail studies to investigate the effects of vegetation on the population density in non-tropical (Hänsel, Walther & Plachter, 1999; Horsák, Škodová & Cernohorsky, 2011; Barrientos, 2000; Barrientos, 2019; Caldwell et al., 2014) and tropical regions (Barrientos, 2000). However, these studies relate the abundance of land snails, either with the general characteristic of vegetation structures (e.g., herbaceous vegetation in Hänsel, Walther & Plachter (1999); sparse herb vegetation in Horsák, Škodová & Cernohorsky (2011); thickness of herbaceous vegetation in Barrientos (2000)) or common plant species in the study sites (e.g., Barrientos, 2000; Caldwell et al., 2014). These studies could not establish proof of causation between specific plant species and the abundance of land snails. Although we could not confirm the in-depth association between the vascular plant species and snail feeding ecology, we were able to identify candidate plants to be included in future experiments. It would be worthwhile to investigate the pH and nutrient content of the plant species as the possible food sources and shelters for the land snail since there was an association between snail abundance and particular plant species.

### Other factors that may affect the distribution and density of land snails

The distribution and density of *P. elephas* may be influenced by factors that were not investigated in this study, such as calcium availability, pH of the substrates, and predators. In the non-limestone forests, calcium availability plays a major role in determining the population density of snails (Gardenfors, 1992; Graveland & Van der Wal, 1996; Skeldon et al., 2007). However, we assumed that calcium availability may not vary significantly because all the plots were located next to the limestone outcrops (Crowther, 1987a); therefore, calcium availability may not be a factor that requires further attention among the plots.

Typically, the pH of the substrates (soil and leaf litter) will be affected by the bedrock, and the variation of pH among the plots on the limestone hills were very small (e.g., Crowther, 1982). However, leaf pH and leaf litter can vary significantly among different plant species (Cornelissen et al., 2011; Tao et al., 2019). However, a study of other land snails in another part of the world suggested that snail density correlated with calcium content and, to a lesser extent, with the pH of the litter layer (Graveland & Van der Wal, 1996).

Lastly, predation pressure could also explain snails’ density distribution patterns (Abramsky et al., 1992; Meyer & Cowie, 2010; Gerlach et al., 2020). Unfortunately, data on population densities and life histories for predators were not available from this study.

## Conclusion

We determined the ecological aspects of *P. elephas* in terms of the habitat, topography, microclimate, and vegetation variables. We also found that ground temperature and a few vascular plant species were positively associated with snail abundance. Although our study was limited by its short duration and the absence of replicate sites on other hills, our findings can be used to formulate testable hypotheses when another population of this snail is found on further sites. After this exploratory study, we suggest a more focused, hypothesis-driven study to determine: (1) how the microclimates variations affect the land snails’ activities during the day and night; (2) the roles of the four vascular plant species that were found associated with living snails as food or shelter.

##  Supplemental Information

10.7717/peerj.11886/supp-1 Supplemental Information 1 Raw data of pilot study and capture-mark-recapture study in the plots

10.7717/peerj.11886/supp-2 Supplemental Information 2 An R script for PCA analysis and plotting chart of microclimate data An R script for principal component analysis (PCA) to assess the degree of habitat heterogeneity among the 17 plots based on the two habitat variables (leaf litter thickness, and canopy cover), four topographic variables (elevation, slope, aspect, and ruggedness index) and two vegetation variables (number of vascular plant individuals and number of vascular plant species). The dataset of these variables can be found in File S4. In addition, the R script for plotting the microclimatic data to examine the variability of the climate data in File S6.

10.7717/peerj.11886/supp-3 Supplemental Information 3 The dataset and the output of the analysis in JASP format for correction tests between the abundance of snails and each of the habitat, vegetation, and topography variables The dataset and the output of the analysis can be viewed by using JASP software version 0.12.2 (JASP Team, 2020).

10.7717/peerj.11886/supp-4 Supplemental Information 4 The dataset for the habitat, topographic, vegetation variables This dataset can be analysed by using an R script of File S2 for principal component analysis (PCA) to assess the degree of habitat heterogeneity among the 17 plots based on the two habitat variables (leaf litter thickness, and canopy cover), four topographic variables (elevation, slope, aspect, and ruggedness index) and two vegetation variables (number of vascular plant individuals and number of vascular plant species).

10.7717/peerj.11886/supp-5 Supplemental Information 5 Principal components analysis (PCA) plots of the third axis with the first and the second axis, respectively, for habitat, topography and vegetation variables for all of the 17 plots in a limestone hill The colour of the plots’ label represents the plot location on the centre, northern and southern parts of the limestone hill. Living *Pollicaria elephas* land snails were not found in any of the plots in the southern and central part of the limestone hills. In contrast, the snails were found in the six of the seven plots on the northern part of the same limestone hill (except A-P14 plot).

10.7717/peerj.11886/supp-6 Supplemental Information 6 Microclimate dataset of the plots This dataset consists of three datasets, namely, soil temperature, ambient temperature and ambient humidity of the plots. The R script in File S2 can be used to reproduce the Figs. 4, 5 and 6.

10.7717/peerj.11886/supp-7 Supplemental Information 7 Correlation between the abundance of land snails *Pollicaria elephas* for each plot with the abundance of the associated four vascular plant species

10.7717/peerj.11886/supp-8 Supplemental Information 8 Soil temperature, air temperature and air humidity variations for every two hours of all the days in July 2018 in the plots A figure and raw data for mean soil temperature, air temperature and air humidity for every two hours of all the days in July 2018 in the plots.

## Additional Information and Declarations

### Data Availability

The following information was supplied regarding data availability: The raw data is available in the Supplemental Files. The dataset and the output of the analysis (File S3) can be viewed by using JASP software version 0.12.2 (JASP Team, 2020).
