# Supplementary material for: Distribution and abundance of the land snail Pollicaria elephas (Gastropoda: Pupinidae) in limestone habitats in Perak, Malaysia
Source: PeerJ. 2021 Jul 28;9:e11886. doi: 10.7717/peerj.11886 (PMC8325424; doi:10.7717/peerj.11886)
Supplement: Supplemental Information 1 [file peerj-09-11886-s001.docx]

**Additional File 1: Table for raw data of pilot study and capture-mark-recapture study in the plots.**

**Table 1.** The individual number of *Pollicaria elephas* collected in the pilot survey that was conducted on 11 May 2018. The three numbers in the parentheses are the breakdown of the demography of the collected snails (juvenile (< 3 whorls), subadult (3 – 5 whorls), adult (with aperture lip)

| **Name and location of the plots** | **Pilot Survey** | |
| --- | --- | --- |
|  | **Number of living snails** | **Number of empty shells** |
| ***Central part of the limestone hill*** | | |
| C-P4 | 0 | 0 |
|  |  |  |
| ***Northern part of the limestone hill*** | | |
| A-P0 | 21 (3,0,18) | Not collected |
| A-Polli2 | 25 (7,0,18) | 7 |
| A-Polli3 | 2 (0,0,2) | 0 |
| A-Polli9 | 22 (3,0,19) | 14 |
| A-Polli7 | 15 (2,0,13) | 24 |
| A-P2 | 8 (3,0,5) | 6 |
| A-P14 | 0 | 0 |
|  |  |  |
| ***Southern part of the limestone hill*** | | |
| B-P7 | 0 | 0 |
| B-P6 | 0 | 0 |
| D-P8 | 0 | 0 |
| D-P11 | 0 | 0 |
| D-P10 | 0 | 0 |
| Intro-P1 | 0 | 0 |
| D-P9 | 0 | 0 |
| D-P12 | 0 | 0 |
| D-P3 | 0 | 0 |

**Table 2.** Data from capture-mark-recapture sessions of *Pollicaria elephas* collected at plot A-Polli2. The three numbers in the parentheses are the breakdown of the demography of the collected snails (juvenile (< 3 whorls), subadult (3 – 5 whorls), adult (with aperture lip)).

|  | Number of snails caught | Number of recaptures | Number of new snails caught | Total number of marked snails |
| --- | --- | --- | --- | --- |
| 1st capture  (9 July 2018) | 18 (2,1,15) | - | - | 18 (2,1,15) |
| 2nd capture  (19 July 2018) | 26 (3,2,21) | 6 (0,1,5) | 20 (3,1,16) | 38 (5,2,31) |
| 3rd capture  (1 August 2018) | 21 (3,2,16) | 14 (0,0,14) | 7 (3,2,2) | 45 (8,4,33) |
| 4th capture  (16 August 2018) | 25 (10,2,13) | 22 (10,2,10) | 3 (0,0,3) | 48 (8,4,36) |

**Table 3.** Data from capture-mark-recapture sessions of *Pollicaria elephas* collected at plot A-Polli9. The three numbers in the parentheses are the breakdown of the demography of the collected snails (juvenile (< 3 whorls), subadult (3 – 5 whorls), adult (with aperture lip)).

|  | Number of snails caught | Number of recaptures | Number of new snails caught | Total number of marked snails |
| --- | --- | --- | --- | --- |
| 1st capture  (9 July 2018) | 16 (0,0,16) | - | - | 16 (0,0,16) |
| 2nd capture  (19 July 2018) | 18 (0,0,18) | 16 (0,0,16) | 2 (0,0,2) | 18 (0,0,18) |
| 3rd capture  (1 August 2018) | 15 (0,0,15) | 12 (0,0,12) | 3 (0,0,3) | 21 (0,0,21) |
| 4th capture  (16 August 2018) | 16 (0,1,15) | 15 (0,0,15) | 1 (0,1,0) | 22 (0,1,21) |

**Table 4.** Data from capture-mark-recapture sessions of *Pollicaria elephas* collected at plot A-Polli7. The three numbers in the parentheses are the breakdown of the demography of the collected snails (juvenile (< 3 whorls), subadult (3 – 5 whorls), adult (with aperture lip)).

|  | Number of snails caught | Number of recaptures | Number of new snails caught | Total number of marked snails |
| --- | --- | --- | --- | --- |
| 1st capture  (9 July 2018) | 16 (1,1,14) | - | - | 16 (1,1,14) |
| 2nd capture  (19 July 2018) | 18 (1,1,16) | 16 (1,1,14) | 2 (0,0,2) | 18 (1,1,16) |
| 3rd capture  (1 August 2018) | 15 (0,0,15) | 13 (0,0,13) | 2 (0,0,2) | 20 (1,1,17) |
| 4th capture  (16 August 2018) | 14 (1,0,13) | 14 (1,0,13) | 0 (0,0,0) | 20 (1,1,17) |

**Table 5.** Data from capture-mark-recapture sessions of *Pollicaria elephas* collected at plot A-Polli3. The three numbers in the parentheses are the breakdown of the demography of the collected snails (juvenile (< 3 whorls), subadult (3 – 5 whorls), adult (with aperture lip)).

|  | Number of snails caught | Number of recaptures | Number of new snails caught | Total number of marked snails |
| --- | --- | --- | --- | --- |
| 1st capture  (9 July 2018) | 1 (0,0,1) | - | - | 1 (0,0,1) |
| 2nd capture  (19 July 2018) | 1 (0,0,1) | 1(0,0,1) | 0 (0,0,0) | 1 (0,0,1) |
| 3rd capture  (1 August 2018) | 1 (0,0,1) | 1 (0,0,1) | 0 (0,0,0) | 1 (0,0,1) |
| 4th capture  (16 August 2018) | 2 (0,0,2) | 1 (0,0,1) | 1 (0,0,1) | 2 (0,0,2) |

**Table 6.** Data from capture-mark-recapture sessions of *Pollicaria elephas* collected at plot A-P2. The three numbers in the parentheses are the breakdown of the demography of the collected snails (juvenile (< 3 whorls), subadult (3 – 5 whorls), adult (with aperture lip)).

|  | Number of snails caught | Number of recaptures | Number of new snails caught | Total number of marked snails |
| --- | --- | --- | --- | --- |
| 1st capture  (9 July 2018) | 7 (2,0,5) | - | - | 7 (2,0,5) |
| 2nd capture  (19 July 2018) | 7 (4,0,3) | 5 (2,0,3) | 2 (2,0,0) | 9 (4,0,5) |
| 3rd capture  (1 August 2018) | 4 (2,0,2) | 2 (0,0,2) | 2 (2,0,0) | 11 (6,0,5) |
