# Supplementary material for: Distribution and abundance of the land snail Pollicaria elephas (Gastropoda: Pupinidae) in limestone habitats in Perak, Malaysia
Source: PeerJ. 2021 Jul 28;9:e11886. doi: 10.7717/peerj.11886 (PMC8325424; doi:10.7717/peerj.11886)
Supplement: Supplemental Information 3 — The dataset and the output of the analysis can be viewed by using JASP software version 0.12.2 (JASP Team, 2020). [file peerj-09-11886-s003.jasp › index.html]

JASP 


# Results

## Correlation

| Kendall's Tau Correlations | | | | | | | | | | | | | | | | | | | | | |
| --- | --- | --- | --- | --- | --- | --- | --- | --- | --- | --- | --- | --- | --- | --- | --- | --- | --- | --- | --- | --- | --- |
| Variable | |  | | Number of living snail | | Canopy Cover (%) | | Leaf litters thickness (cm) | | Elevation (meters) | | Aspect (counterclockwise in degrees from 0 (due north) to 360 (again due north)) | | Ruggedness Index | | Slope (in degrees) | | Total identified vascular plant individual for each plot | | Total identified vascular species for each plot | |
| 1. Number of living snail |  | Kendall's Tau B |  | — |  |  |  |  |  |  |  |  |  |  |  |  |  |  |  |  |  |
|  |  | p-value |  | — |  |  |  |  |  |  |  |  |  |  |  |  |  |  |  |  |  |
| 2. Canopy Cover (%) |  | Kendall's Tau B |  | 0.261 |  | — |  |  |  |  |  |  |  |  |  |  |  |  |  |  |  |
|  |  | p-value |  | 0.216 |  | — |  |  |  |  |  |  |  |  |  |  |  |  |  |  |  |
| 3. Leaf litters thickness (cm) |  | Kendall's Tau B |  | 0.154 |  | -0.077 |  | — |  |  |  |  |  |  |  |  |  |  |  |  |  |
|  |  | p-value |  | 0.436 |  | 0.692 |  | — |  |  |  |  |  |  |  |  |  |  |  |  |  |
| 4. Elevation (meters) |  | Kendall's Tau B |  | 0.183 |  | -0.238 |  | 0.000 |  | — |  |  |  |  |  |  |  |  |  |  |  |
|  |  | p-value |  | 0.355 |  | 0.218 |  | 1.000 |  | — |  |  |  |  |  |  |  |  |  |  |  |
| 5. Aspect (counterclockwise in degrees from 0 (due north) to 360 (again due north)) |  | Kendall's Tau B |  | 0.086 |  | -0.202 |  | 0.230 |  | 0.141 |  | — |  |  |  |  |  |  |  |  |  |
|  |  | p-value |  | 0.662 |  | 0.292 |  | 0.200 |  | 0.432 |  | — |  |  |  |  |  |  |  |  |  |
| 6. Ruggedness Index |  | Kendall's Tau B |  | 0.092 |  | 0.097 |  | 0.134 |  | 0.011 |  | 0.253 |  | — |  |  |  |  |  |  |  |
|  |  | p-value |  | 0.669 |  | 0.646 |  | 0.509 |  | 1.000 |  | 0.233 |  | — |  |  |  |  |  |  |  |
| 7. Slope (in degrees) |  | Kendall's Tau B |  | 0.241 |  | -0.102 |  | 0.015 |  | 0.353 |  | 0.364 | \* | 0.956 | \*\*\* | — |  |  |  |  |  |
|  |  | p-value |  | 0.224 |  | 0.597 |  | 0.934 |  | 0.051 |  | 0.043 |  | < .001 |  | — |  |  |  |  |  |
| 8. Total identified vascular plant individual for each plot |  | Kendall's Tau B |  | -0.186 |  | 0.060 |  | 0.252 |  | 0.183 |  | 0.083 |  | 0.158 |  | 0.198 |  | — |  |  |  |
|  |  | p-value |  | 0.353 |  | 0.757 |  | 0.170 |  | 0.318 |  | 0.648 |  | 0.440 |  | 0.279 |  | — |  |  |  |
| 9. Total identified vascular species for each plot |  | Kendall's Tau B |  | 0.128 |  | 0.096 |  | 0.169 |  | 0.400 | \* | 0.145 |  | 0.148 |  | 0.323 |  | 0.547 | \*\* | — |  |
|  |  | p-value |  | 0.525 |  | 0.627 |  | 0.360 |  | 0.030 |  | 0.430 |  | 0.472 |  | 0.080 |  | 0.003 |  | — |  |
|  | | | | | | | | | | | | | | | | | | | | | |
|  |  |  |  |  |  |  |  |  |  |  |  |  |  |  |  |  |  |  |  |  |  |
| --- | --- | --- | --- | --- | --- | --- | --- | --- | --- | --- | --- | --- | --- | --- | --- | --- | --- | --- | --- | --- | --- |
| \* p < .05, \*\* p < .01, \*\*\* p < .001 | | | | | | | | | | | | | | | | | | | | | |

### Assumption checks

| Shapiro-Wilk Test for Multivariate Normality | | | |
| --- | --- | --- | --- |
| Shapiro-Wilk | | p | |
| 0.567 |  | < .001 |  |
|  | | | |

### Correlation plot

### Kendall's tau B heatmap

## Bayesian Correlation

| Bayesian Kendall's Tau Correlations | | | | | | | | | | | | | | | | | | | | | |
| --- | --- | --- | --- | --- | --- | --- | --- | --- | --- | --- | --- | --- | --- | --- | --- | --- | --- | --- | --- | --- | --- |
| Variable | |  | | Number of living snail | | Canopy Cover (%) | | Leaf litters thickness (cm) | | Elevation (meters) | | Aspect (counterclockwise in degrees from 0 (due north) to 360 (again due north)) | | Ruggedness Index | | Slope (in degrees) | | Total identified vascular plant individual for each plot | | Total identified vascular species for each plot | |
| 1. Number of living snail |  | Kendall's tau |  | — |  |  |  |  |  |  |  |  |  |  |  |  |  |  |  |  |  |
|  |  | BF₁₀ |  | — |  |  |  |  |  |  |  |  |  |  |  |  |  |  |  |  |  |
| 2. Canopy Cover (%) |  | Kendall's tau |  | 0.261 |  | — |  |  |  |  |  |  |  |  |  |  |  |  |  |  |  |
|  |  | BF₁₀ |  | 0.840 |  | — |  |  |  |  |  |  |  |  |  |  |  |  |  |  |  |
| 3. Leaf litters thickness (cm) |  | Kendall's tau |  | 0.154 |  | -0.077 |  | — |  |  |  |  |  |  |  |  |  |  |  |  |  |
|  |  | BF₁₀ |  | 0.437 |  | 0.336 |  | — |  |  |  |  |  |  |  |  |  |  |  |  |  |
| 4. Elevation (meters) |  | Kendall's tau |  | 0.183 |  | -0.238 |  | 0.000 |  | — |  |  |  |  |  |  |  |  |  |  |  |
|  |  | BF₁₀ |  | 0.504 |  | 0.708 |  | 0.308 |  | — |  |  |  |  |  |  |  |  |  |  |  |
| 5. Aspect (counterclockwise in degrees from 0 (due north) to 360 (again due north)) |  | Kendall's tau |  | 0.086 |  | -0.202 |  | 0.230 |  | 0.141 |  | — |  |  |  |  |  |  |  |  |  |
|  |  | BF₁₀ |  | 0.343 |  | 0.560 |  | 0.672 |  | 0.413 |  | — |  |  |  |  |  |  |  |  |  |
| 6. Ruggedness Index |  | Kendall's tau |  | 0.092 |  | 0.097 |  | 0.134 |  | 0.011 |  | 0.253 |  | — |  |  |  |  |  |  |  |
|  |  | BF₁₀ |  | 0.372 |  | 0.376 |  | 0.414 |  | 0.338 |  | 0.699 |  | — |  |  |  |  |  |  |  |
| 7. Slope (in degrees) |  | Kendall's tau |  | 0.241 |  | -0.102 |  | 0.015 |  | 0.353 |  | 0.364 |  | 0.956 | \*\*\* | — |  |  |  |  |  |
|  |  | BF₁₀ |  | 0.721 |  | 0.359 |  | 0.309 |  | 1.917 |  | 2.149 |  | 7595.004 |  | — |  |  |  |  |  |
| 8. Total identified vascular plant individual for each plot |  | Kendall's tau |  | -0.186 |  | 0.060 |  | 0.252 |  | 0.183 |  | 0.083 |  | 0.158 |  | 0.198 |  | — |  |  |  |
|  |  | BF₁₀ |  | 0.512 |  | 0.325 |  | 0.781 |  | 0.504 |  | 0.341 |  | 0.449 |  | 0.549 |  | — |  |  |  |
| 9. Total identified vascular species for each plot |  | Kendall's tau |  | 0.128 |  | 0.096 |  | 0.169 |  | 0.400 |  | 0.145 |  | 0.148 |  | 0.323 |  | 0.547 | \* | — |  |
|  |  | BF₁₀ |  | 0.392 |  | 0.353 |  | 0.469 |  | 3.201 |  | 0.419 |  | 0.433 |  | 1.422 |  | 23.979 |  | — |  |
|  | | | | | | | | | | | | | | | | | | | | | |
|  |  |  |  |  |  |  |  |  |  |  |  |  |  |  |  |  |  |  |  |  |  |
| --- | --- | --- | --- | --- | --- | --- | --- | --- | --- | --- | --- | --- | --- | --- | --- | --- | --- | --- | --- | --- | --- |
| \*  BF₁₀ > 10, \*\* BF₁₀ > 30, \*\*\* BF₁₀ > 100 | | | | | | | | | | | | | | | | | | | | | |

### Bayesian Correlation Pairwise Plots

#### Number of living snail - Canopy Cover (%)

##### Scatterplot

##### Bayes Factor Robustness Check

#### Number of living snail - Leaf litters thickness (cm)

##### Scatterplot

##### Bayes Factor Robustness Check

#### Number of living snail - Elevation (meters)

##### Scatterplot

##### Bayes Factor Robustness Check

#### Number of living snail - Aspect (counterclockwise in degrees from 0 (due north) to 360 (again due north))

##### Scatterplot

##### Bayes Factor Robustness Check

#### Number of living snail - Ruggedness Index

##### Scatterplot

##### Bayes Factor Robustness Check

#### Number of living snail - Slope (in degrees)

##### Scatterplot

##### Bayes Factor Robustness Check

#### Number of living snail - Total identified vascular plant individual for each plot

##### Scatterplot

##### Bayes Factor Robustness Check

#### Number of living snail - Total identified vascular species for each plot

##### Scatterplot

##### Bayes Factor Robustness Check
