# Supplementary figures and images for: Distribution and abundance of the land snail Pollicaria elephas (Gastropoda: Pupinidae) in limestone habitats in Perak, Malaysia
Source: PeerJ. 2021 Jul 28;9:e11886. doi: 10.7717/peerj.11886 (PMC8325424; doi:10.7717/peerj.11886)

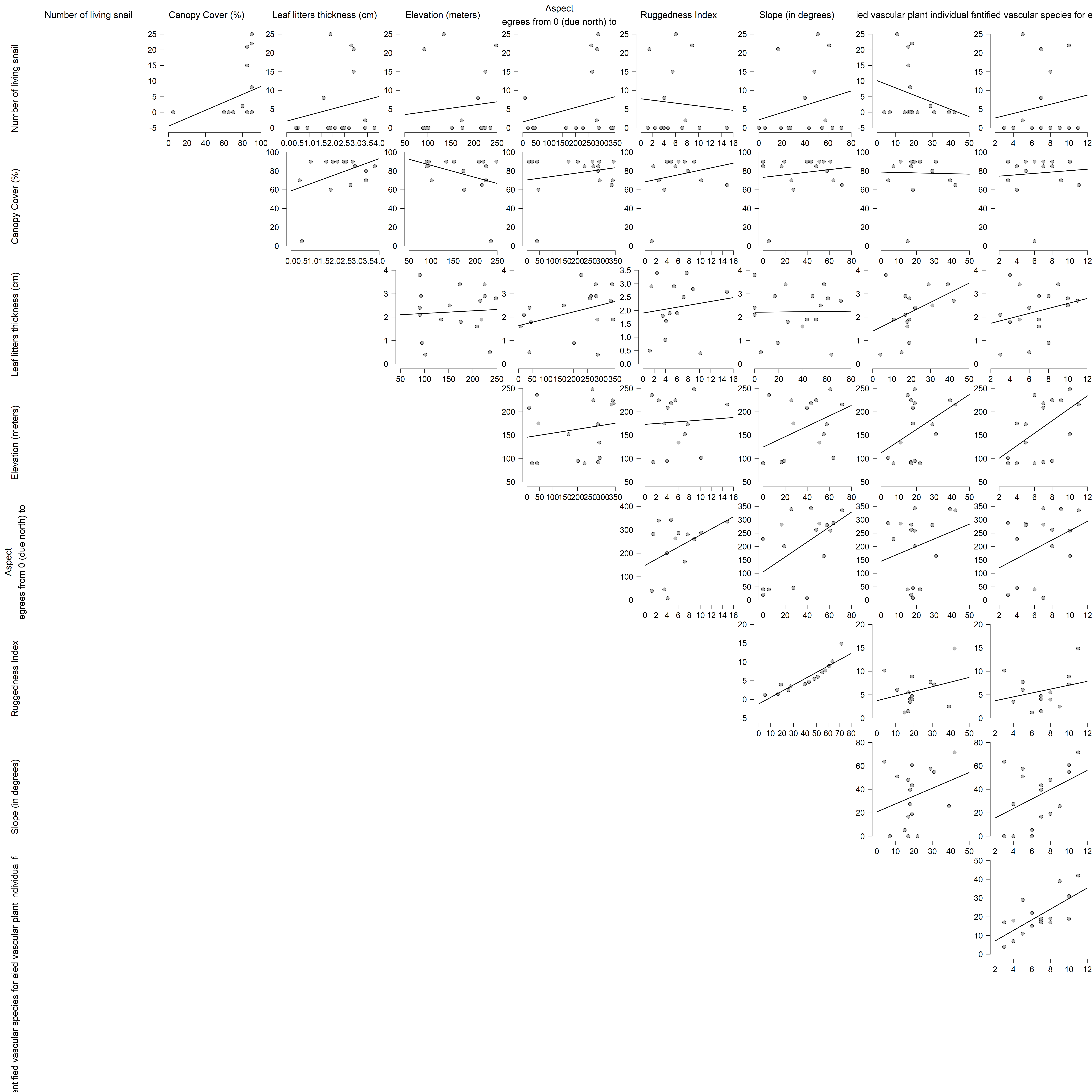

Supplement: Supplemental Information 3 — The dataset and the output of the analysis can be viewed by using JASP software version 0.12.2 (JASP Team, 2020). [file peerj-09-11886-s003.jasp › resources/0/_22_t1656841138.png]

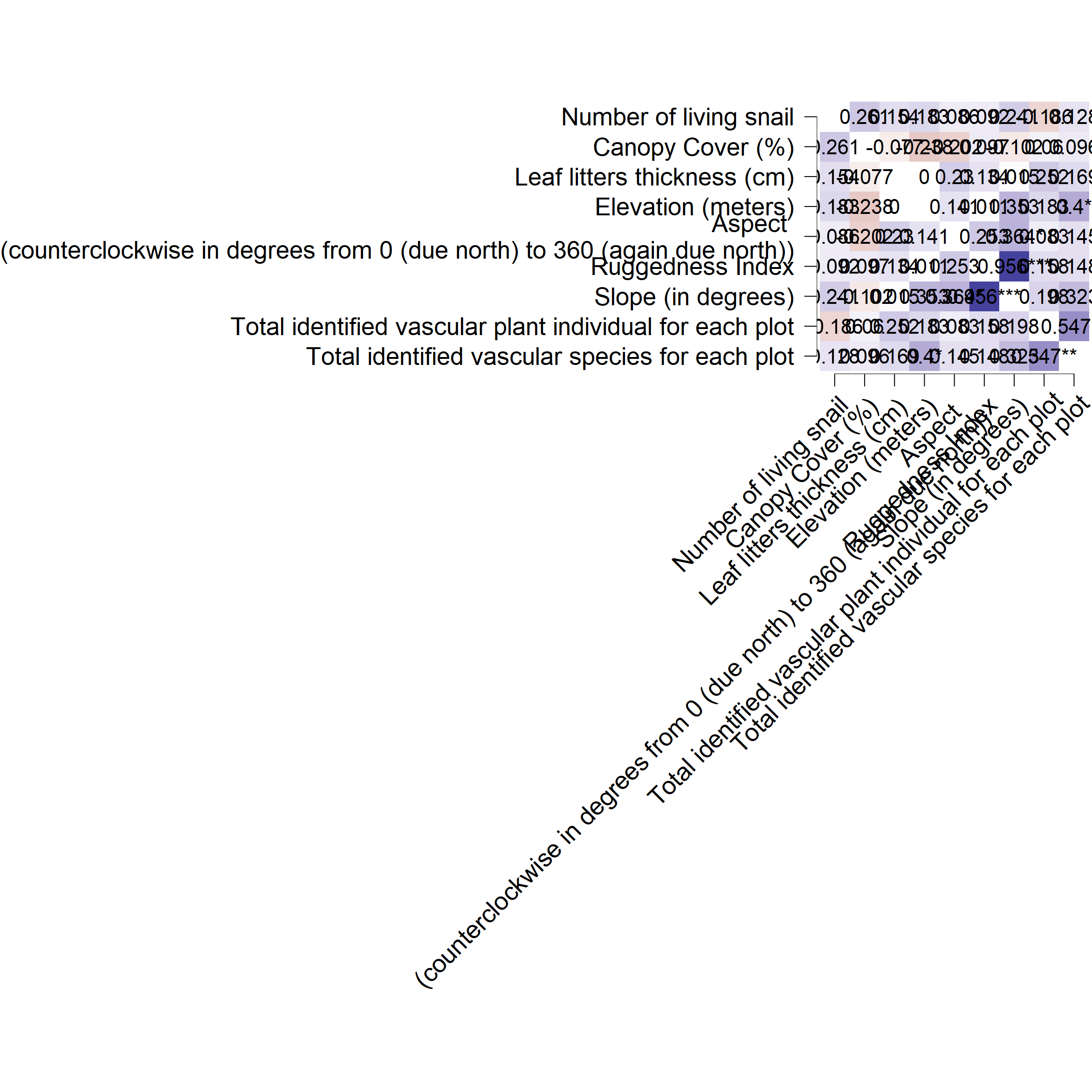

Supplement: Supplemental Information 3 — The dataset and the output of the analysis can be viewed by using JASP software version 0.12.2 (JASP Team, 2020). [file peerj-09-11886-s003.jasp › resources/0/_23_t1656851636.png]

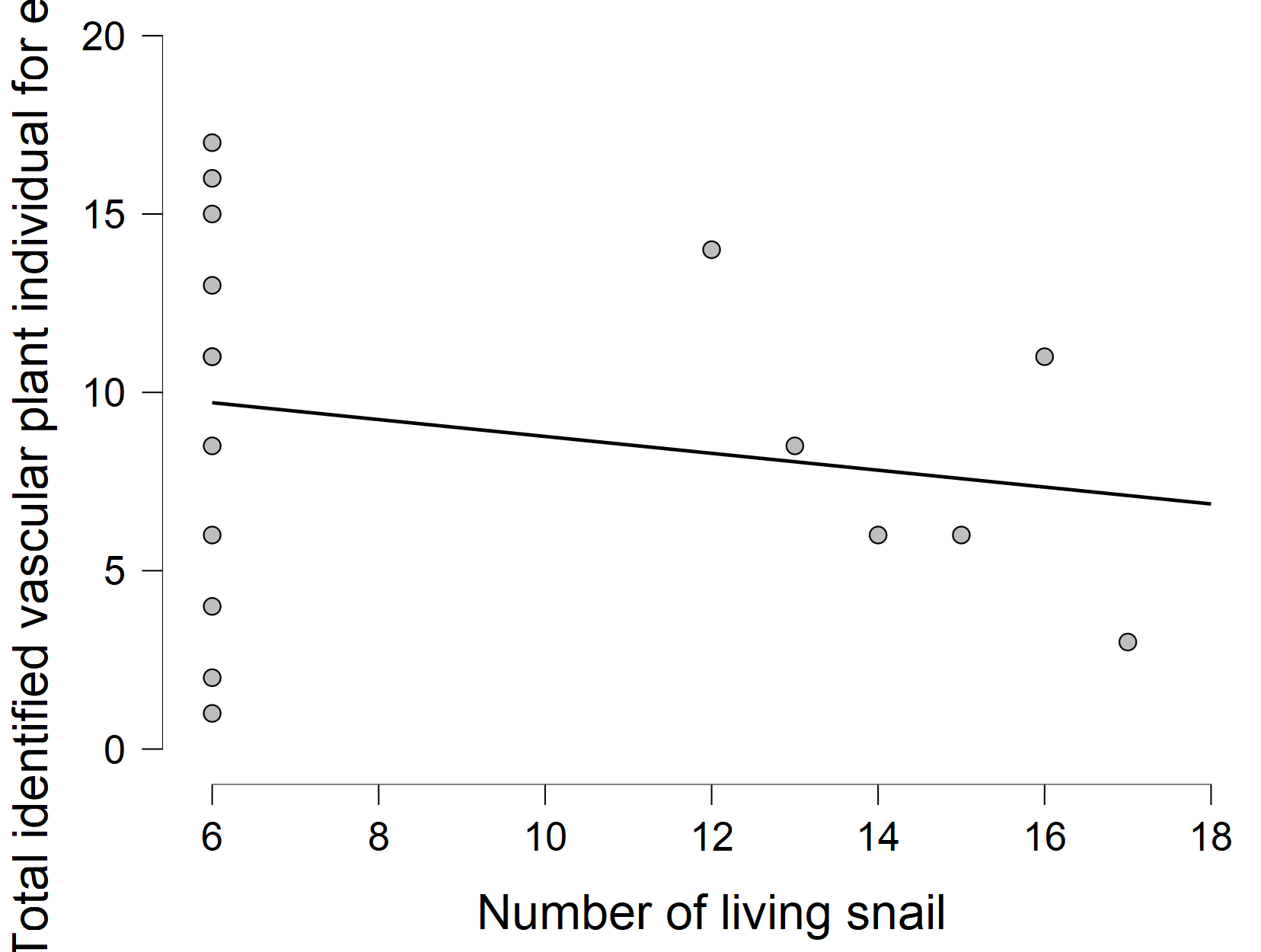

Supplement: Supplemental Information 3 — The dataset and the output of the analysis can be viewed by using JASP software version 0.12.2 (JASP Team, 2020). [file peerj-09-11886-s003.jasp › resources/1/_10_t1656678193.png]

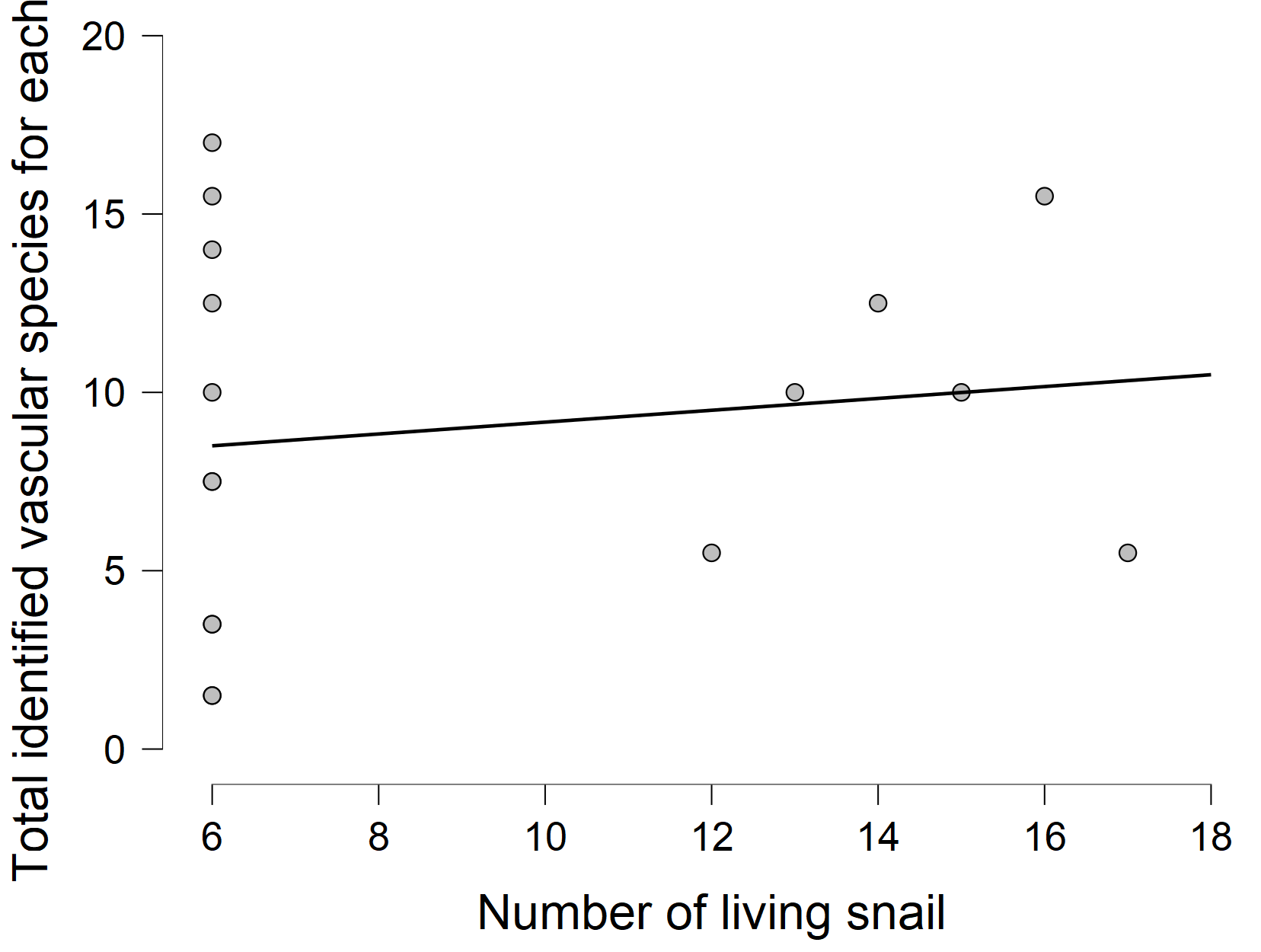

Supplement: Supplemental Information 3 — The dataset and the output of the analysis can be viewed by using JASP software version 0.12.2 (JASP Team, 2020). [file peerj-09-11886-s003.jasp › resources/1/_11_t1656680179.png]

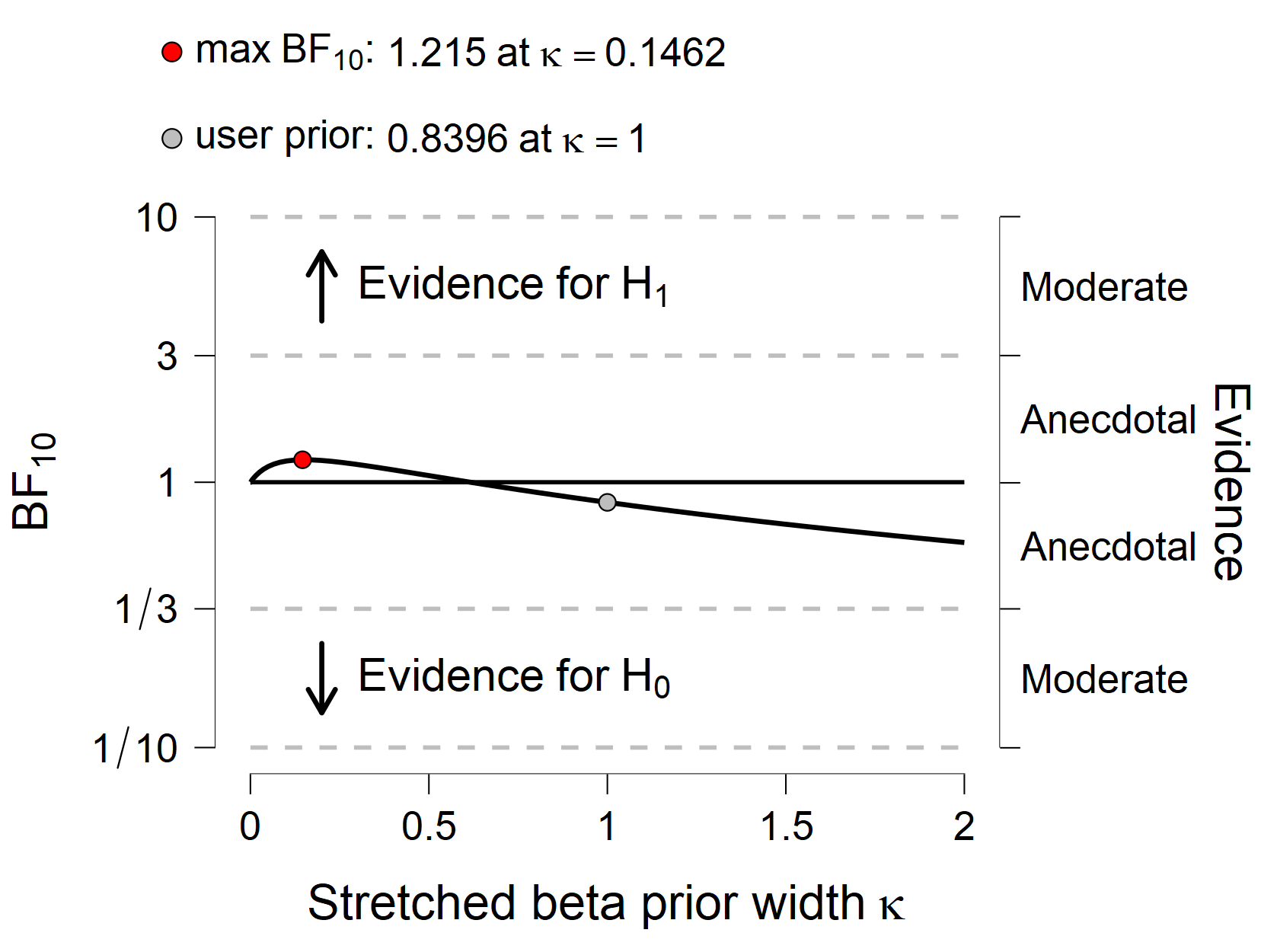

Supplement: Supplemental Information 3 — The dataset and the output of the analysis can be viewed by using JASP software version 0.12.2 (JASP Team, 2020). [file peerj-09-11886-s003.jasp › resources/1/_12_t1656709929.png]

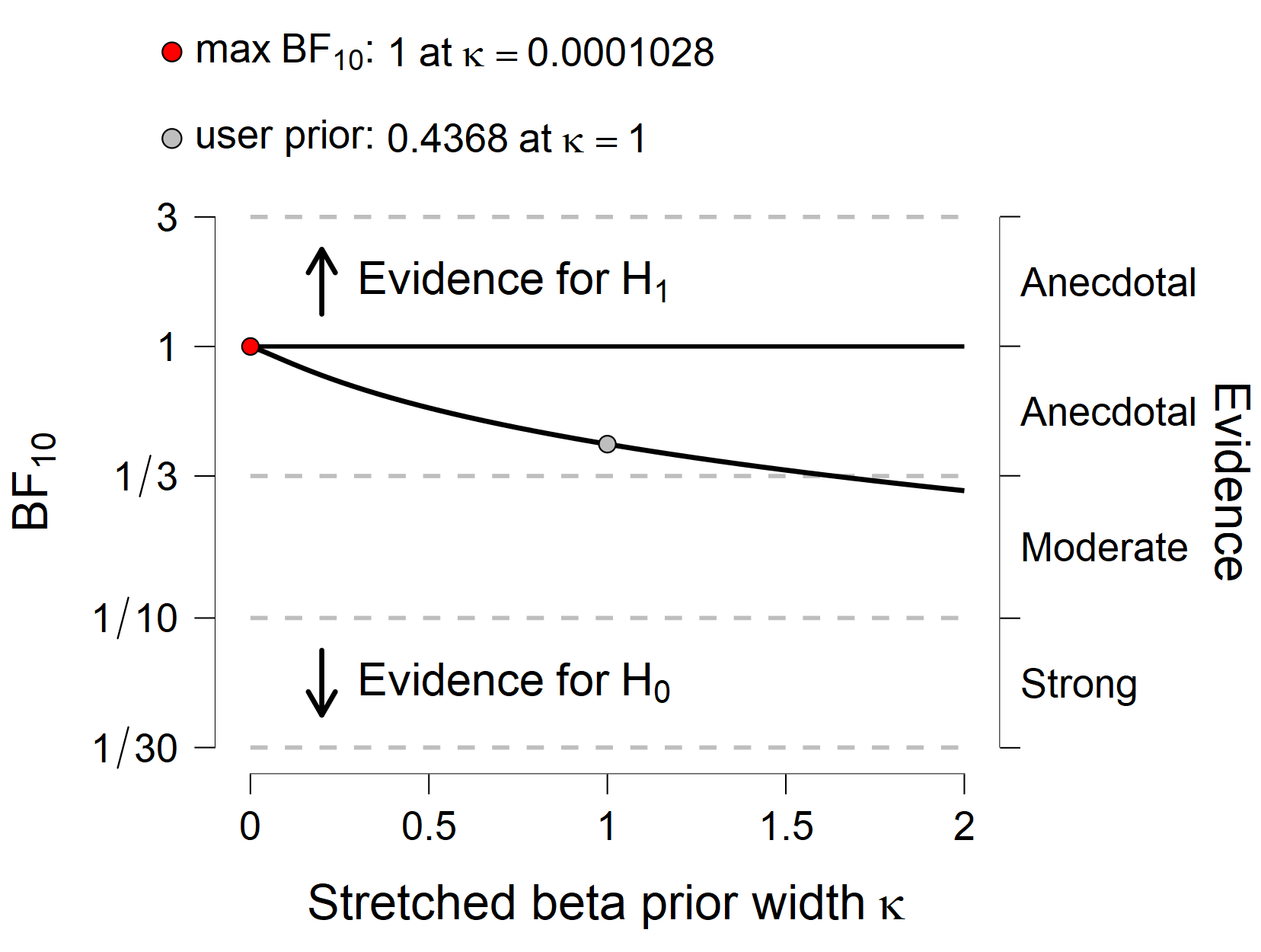

Supplement: Supplemental Information 3 — The dataset and the output of the analysis can be viewed by using JASP software version 0.12.2 (JASP Team, 2020). [file peerj-09-11886-s003.jasp › resources/1/_13_t1656711134.png]

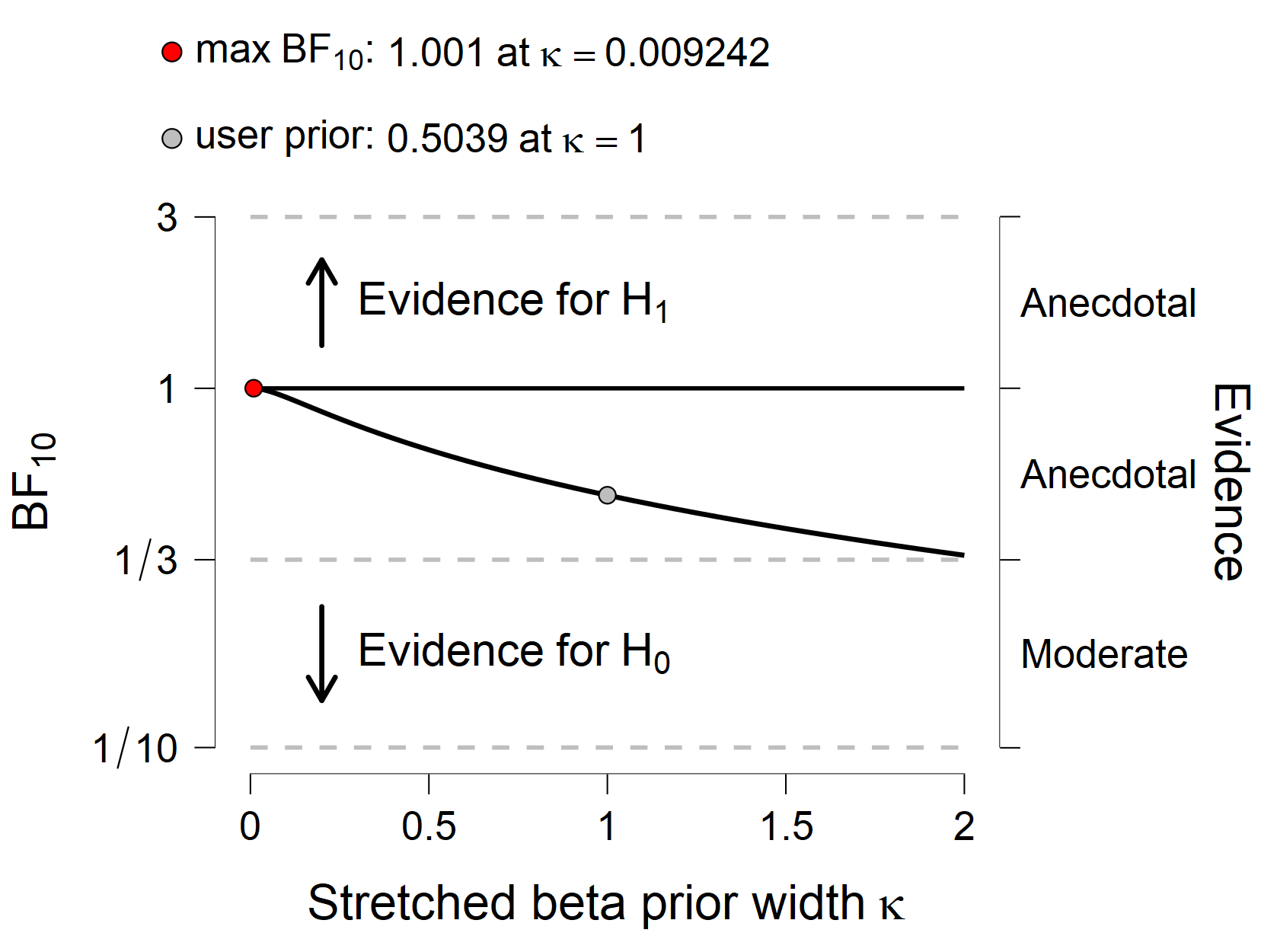

Supplement: Supplemental Information 3 — The dataset and the output of the analysis can be viewed by using JASP software version 0.12.2 (JASP Team, 2020). [file peerj-09-11886-s003.jasp › resources/1/_14_t1656712172.png]

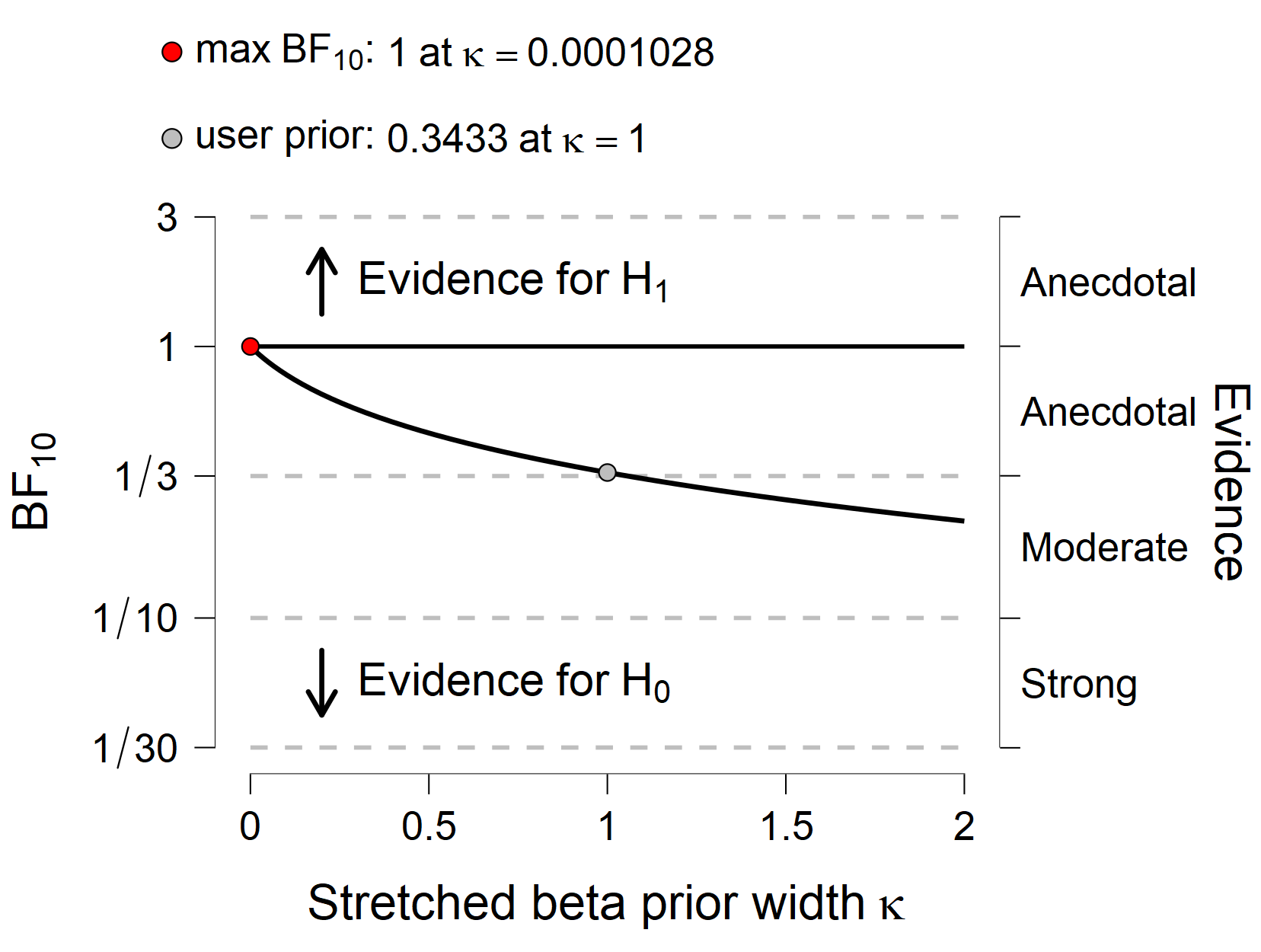

Supplement: Supplemental Information 3 — The dataset and the output of the analysis can be viewed by using JASP software version 0.12.2 (JASP Team, 2020). [file peerj-09-11886-s003.jasp › resources/1/_15_t1656712859.png]

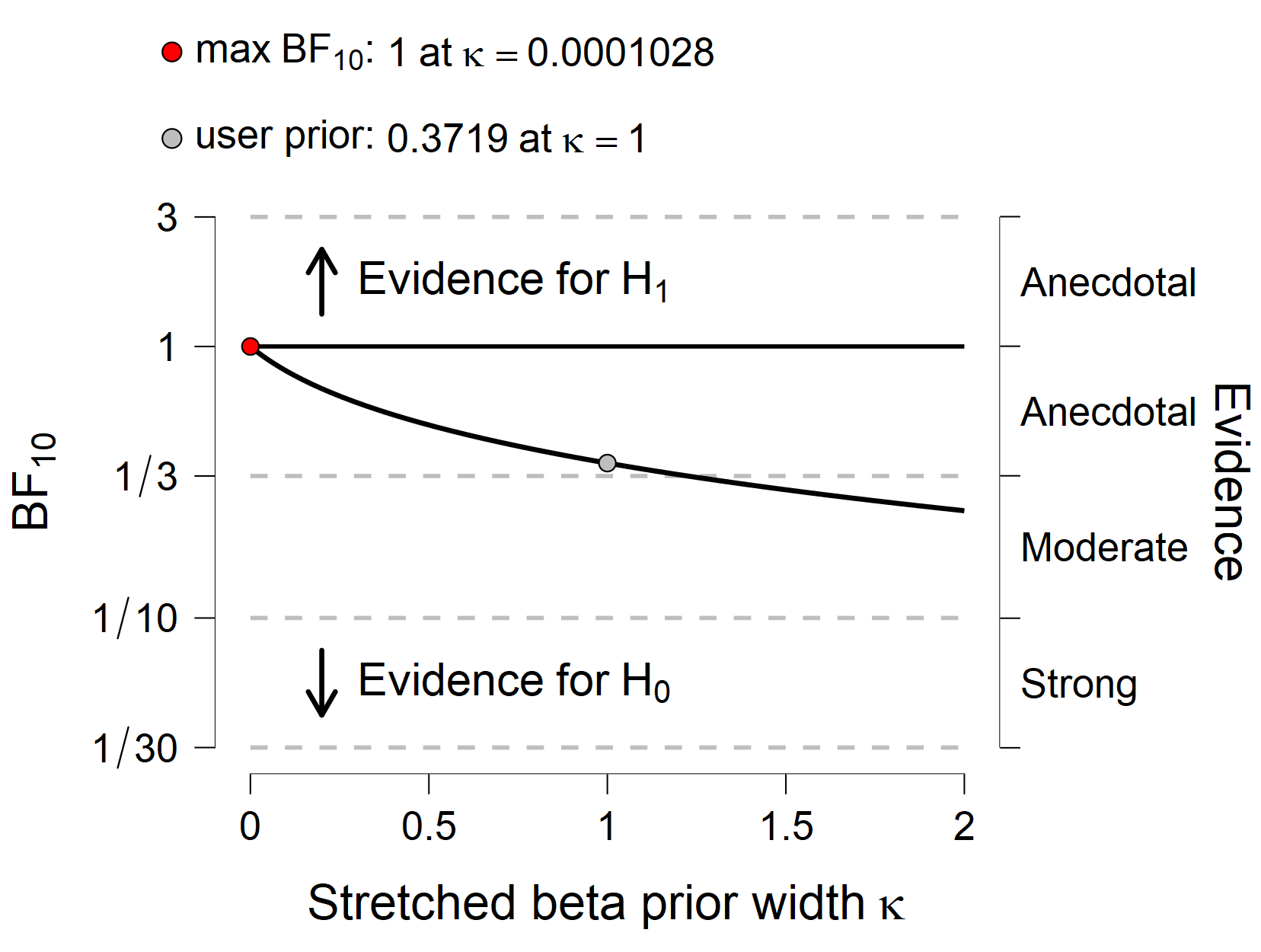

Supplement: Supplemental Information 3 — The dataset and the output of the analysis can be viewed by using JASP software version 0.12.2 (JASP Team, 2020). [file peerj-09-11886-s003.jasp › resources/1/_16_t1656713571.png]

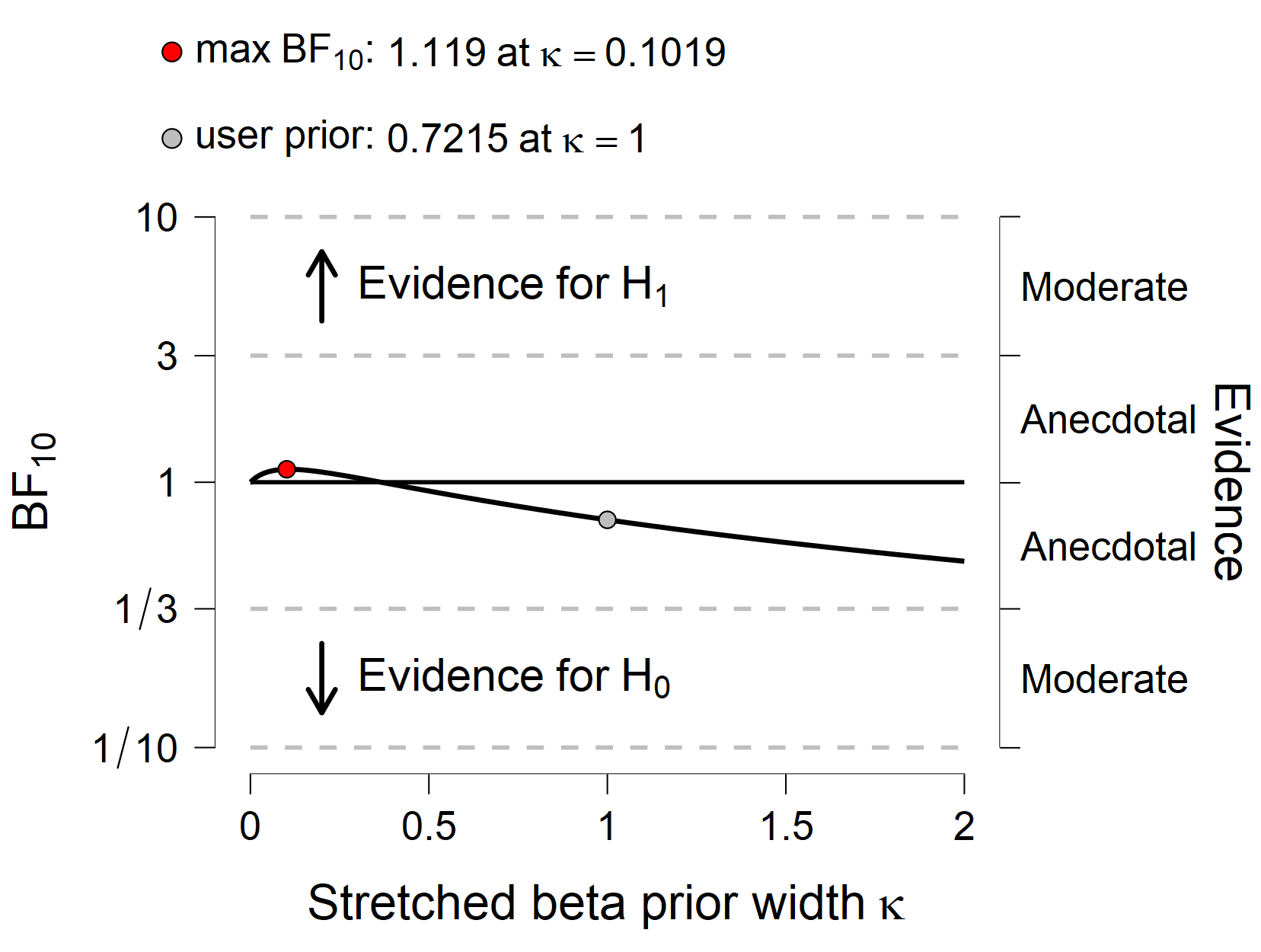

Supplement: Supplemental Information 3 — The dataset and the output of the analysis can be viewed by using JASP software version 0.12.2 (JASP Team, 2020). [file peerj-09-11886-s003.jasp › resources/1/_17_t1656714394.png]

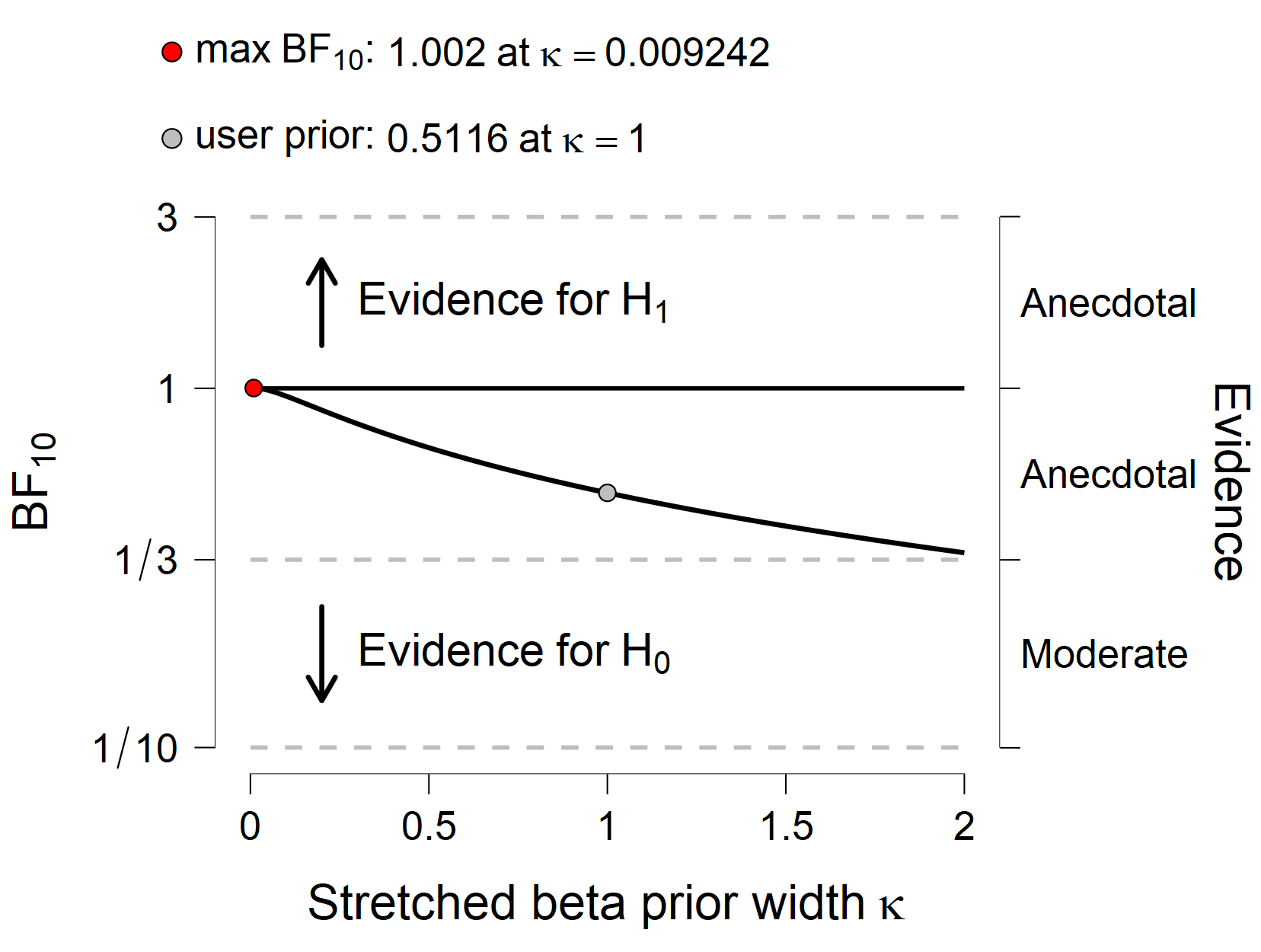

Supplement: Supplemental Information 3 — The dataset and the output of the analysis can be viewed by using JASP software version 0.12.2 (JASP Team, 2020). [file peerj-09-11886-s003.jasp › resources/1/_18_t1656715065.png]

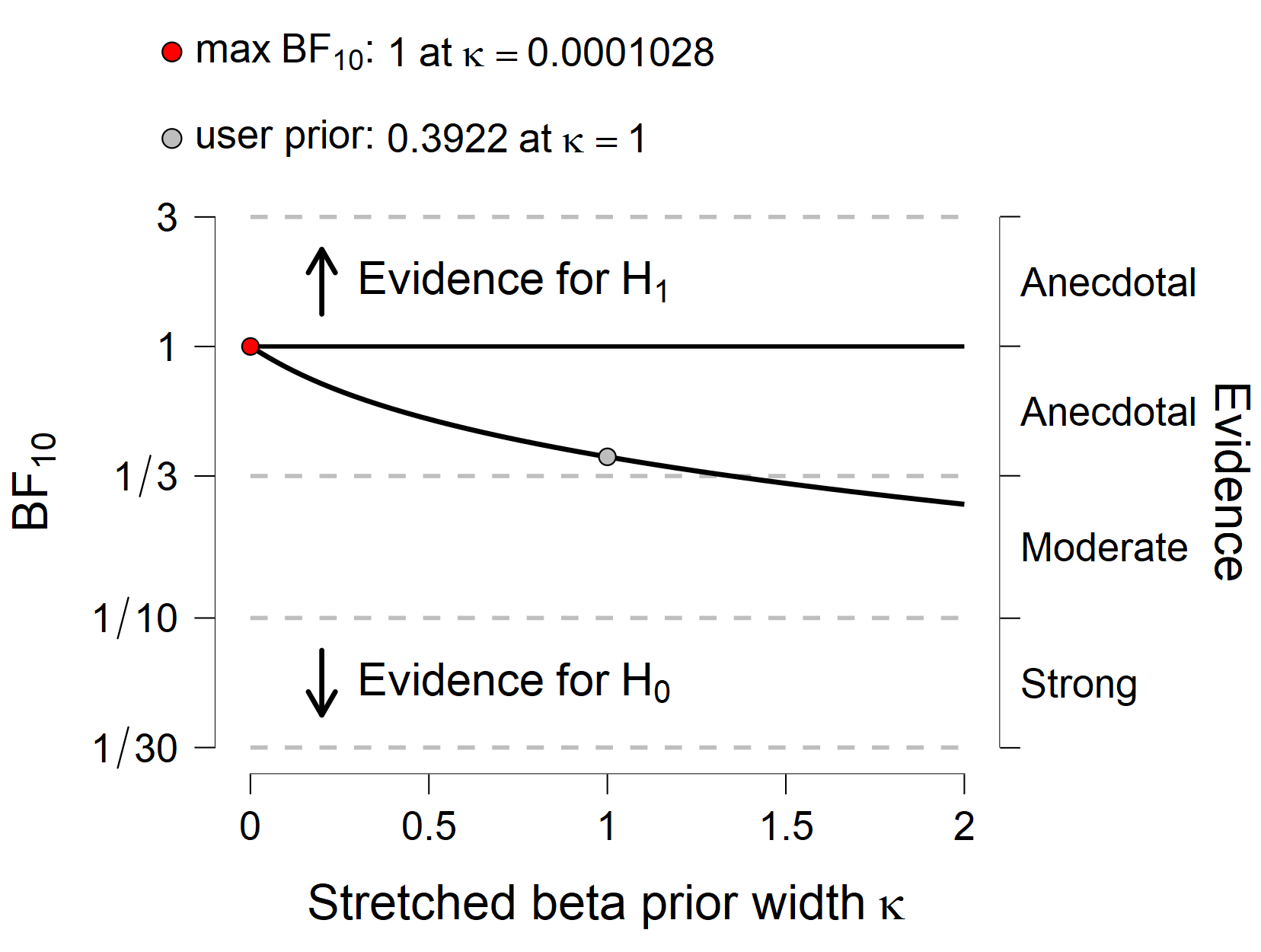

Supplement: Supplemental Information 3 — The dataset and the output of the analysis can be viewed by using JASP software version 0.12.2 (JASP Team, 2020). [file peerj-09-11886-s003.jasp › resources/1/_19_t1656715686.png]

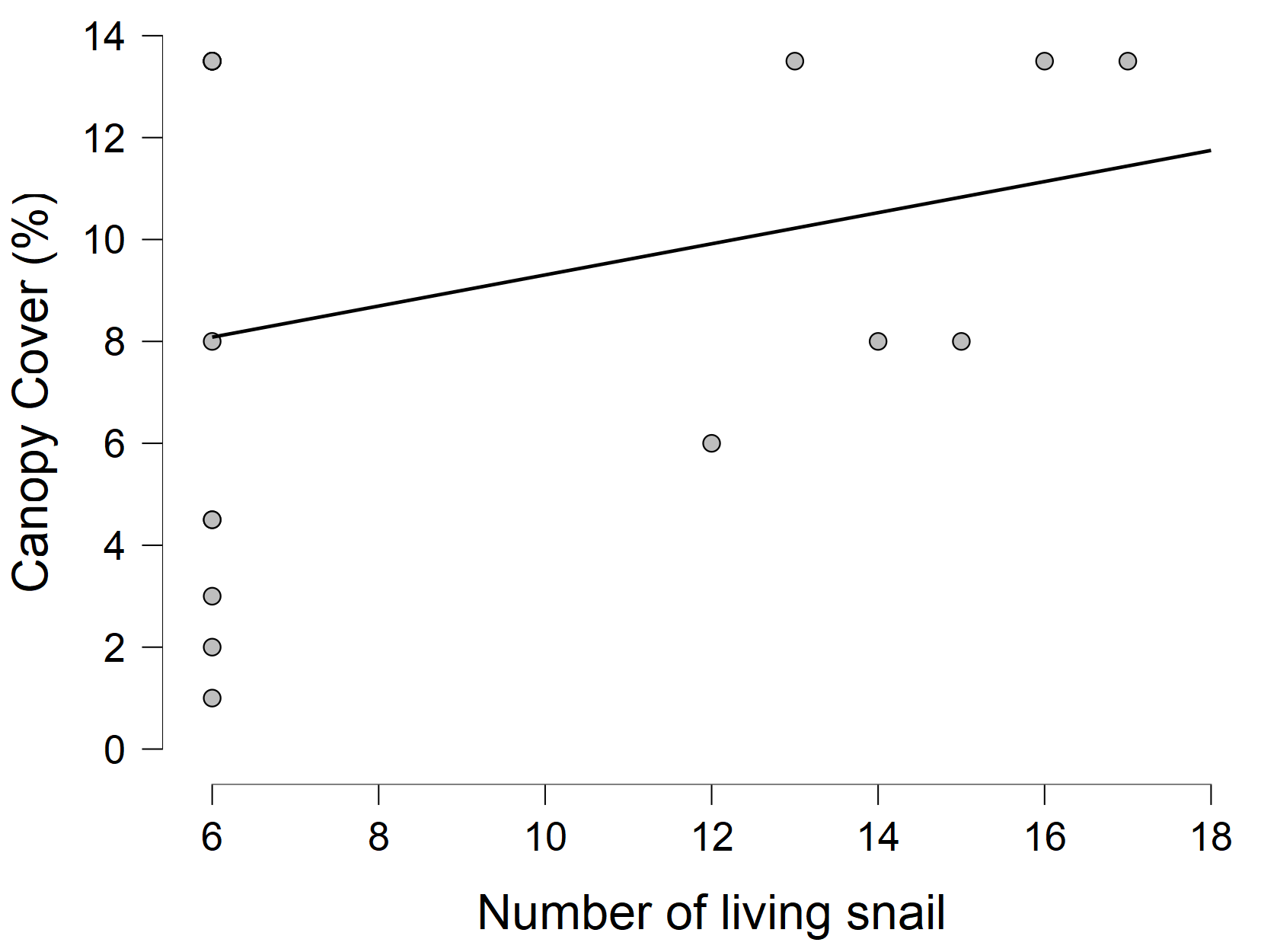

Supplement: Supplemental Information 3 — The dataset and the output of the analysis can be viewed by using JASP software version 0.12.2 (JASP Team, 2020). [file peerj-09-11886-s003.jasp › resources/1/_4_t1656657073.png]

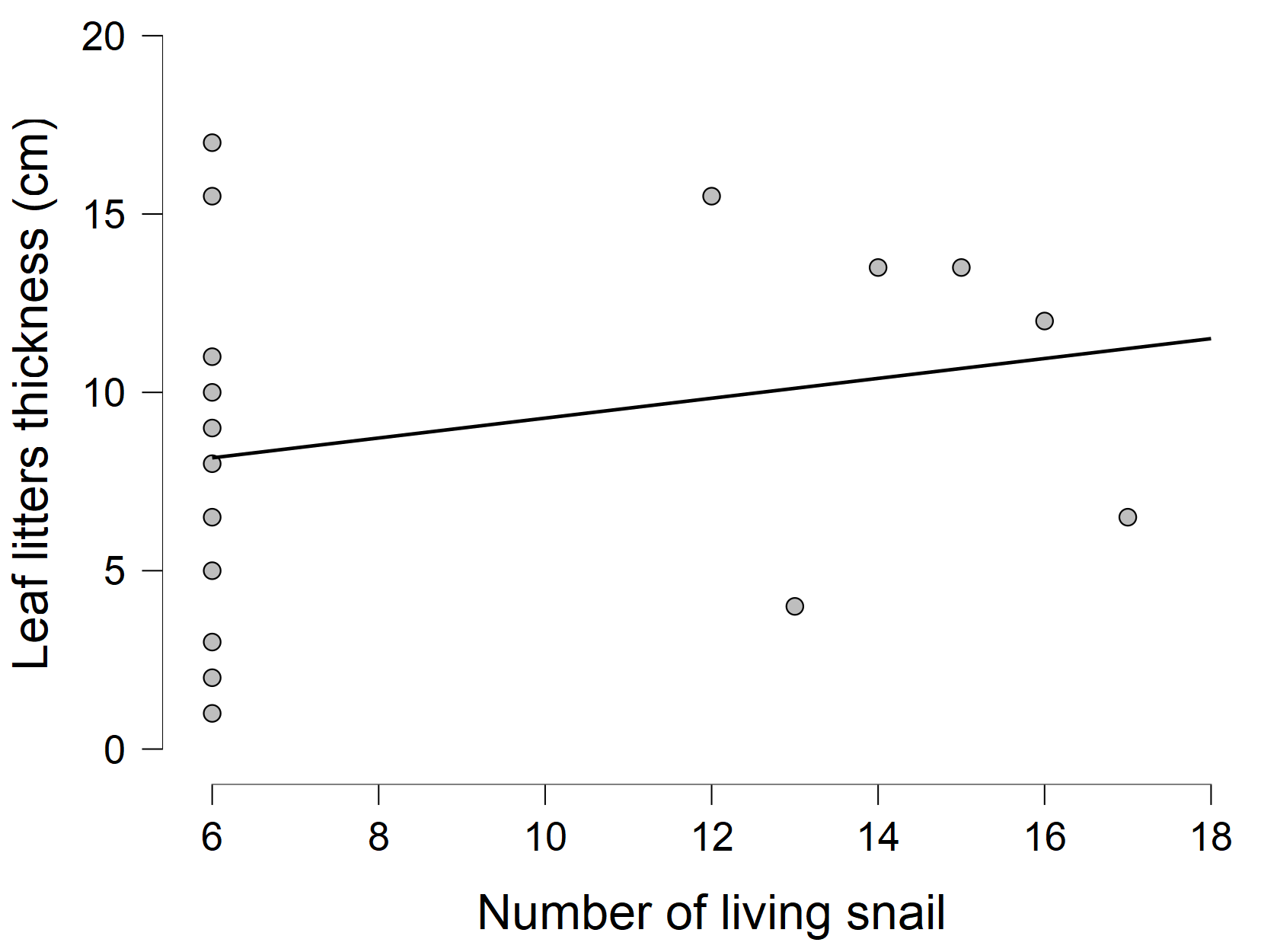

Supplement: Supplemental Information 3 — The dataset and the output of the analysis can be viewed by using JASP software version 0.12.2 (JASP Team, 2020). [file peerj-09-11886-s003.jasp › resources/1/_5_t1656659804.png]

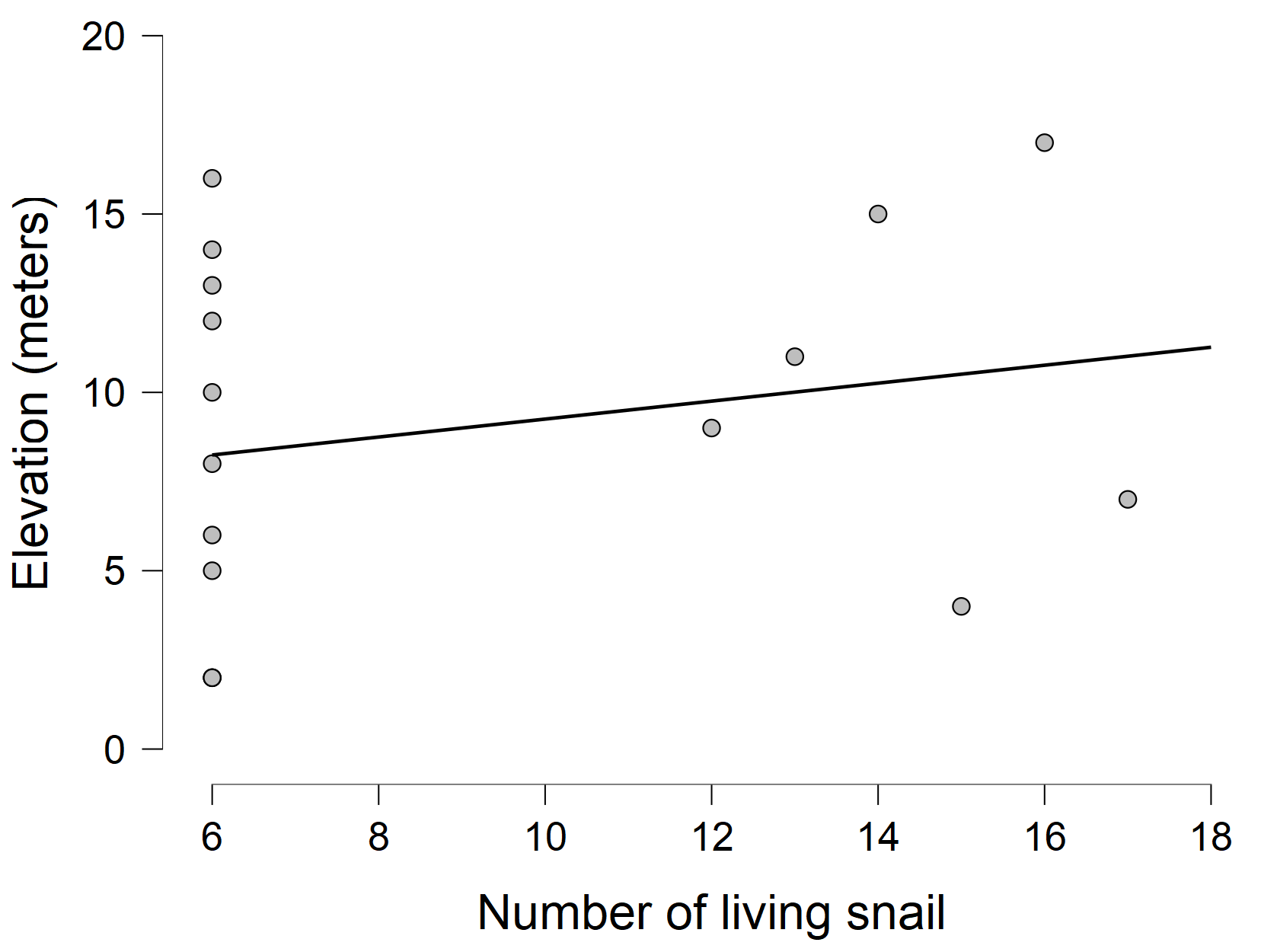

Supplement: Supplemental Information 3 — The dataset and the output of the analysis can be viewed by using JASP software version 0.12.2 (JASP Team, 2020). [file peerj-09-11886-s003.jasp › resources/1/_6_t1656662020.png]

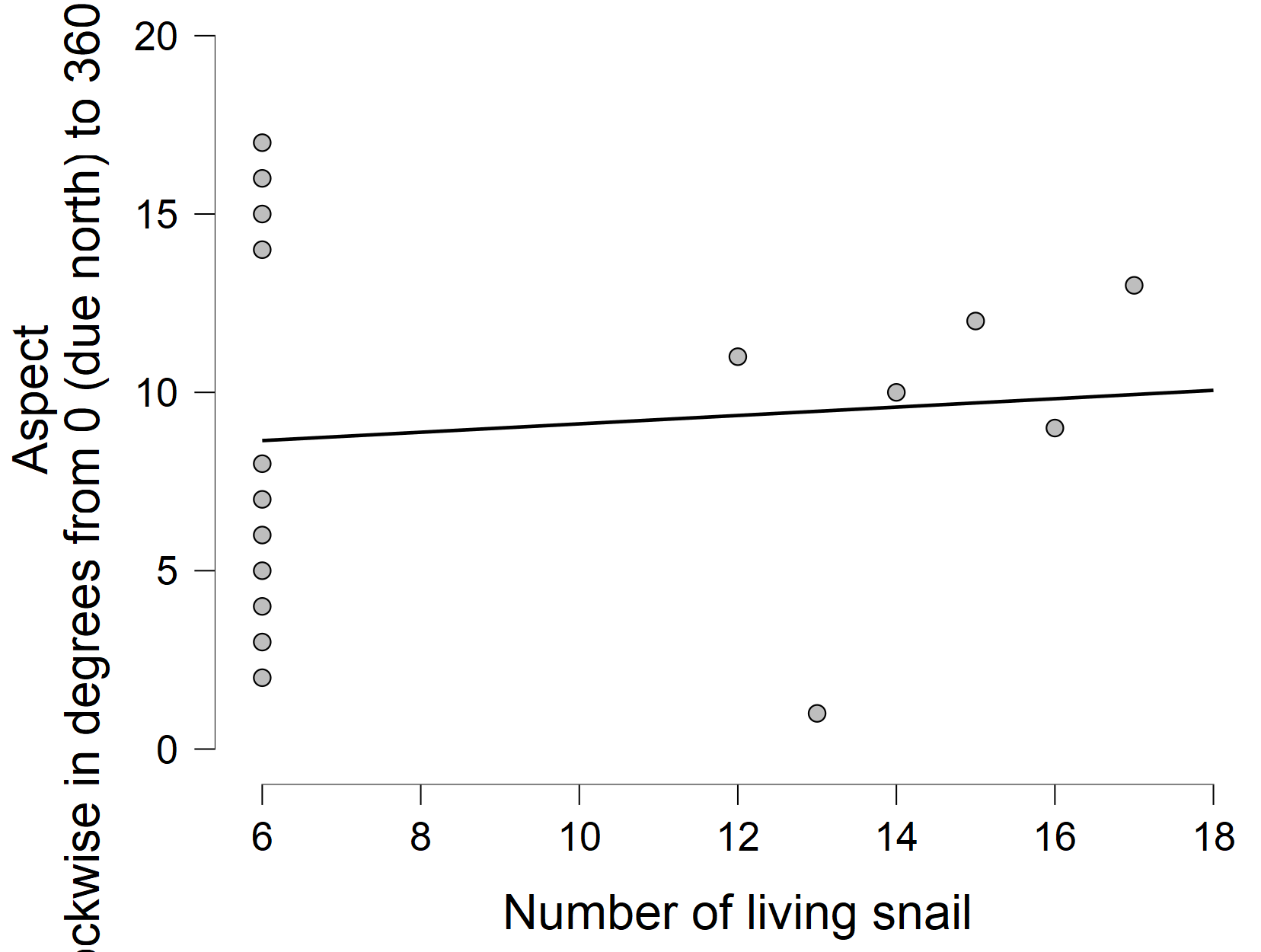

Supplement: Supplemental Information 3 — The dataset and the output of the analysis can be viewed by using JASP software version 0.12.2 (JASP Team, 2020). [file peerj-09-11886-s003.jasp › resources/1/_7_t1656666488.png]

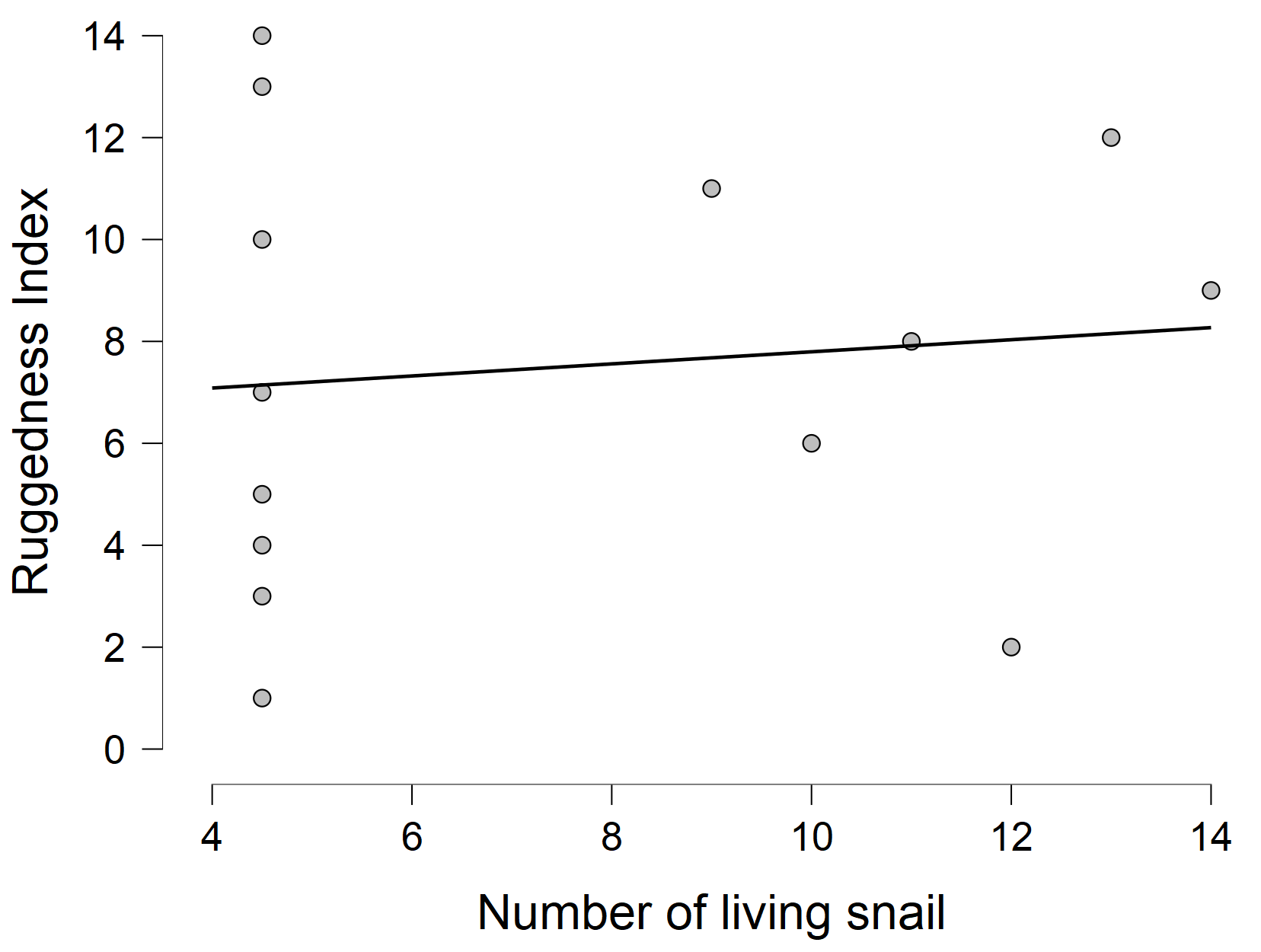

Supplement: Supplemental Information 3 — The dataset and the output of the analysis can be viewed by using JASP software version 0.12.2 (JASP Team, 2020). [file peerj-09-11886-s003.jasp › resources/1/_8_t1656671053.png]

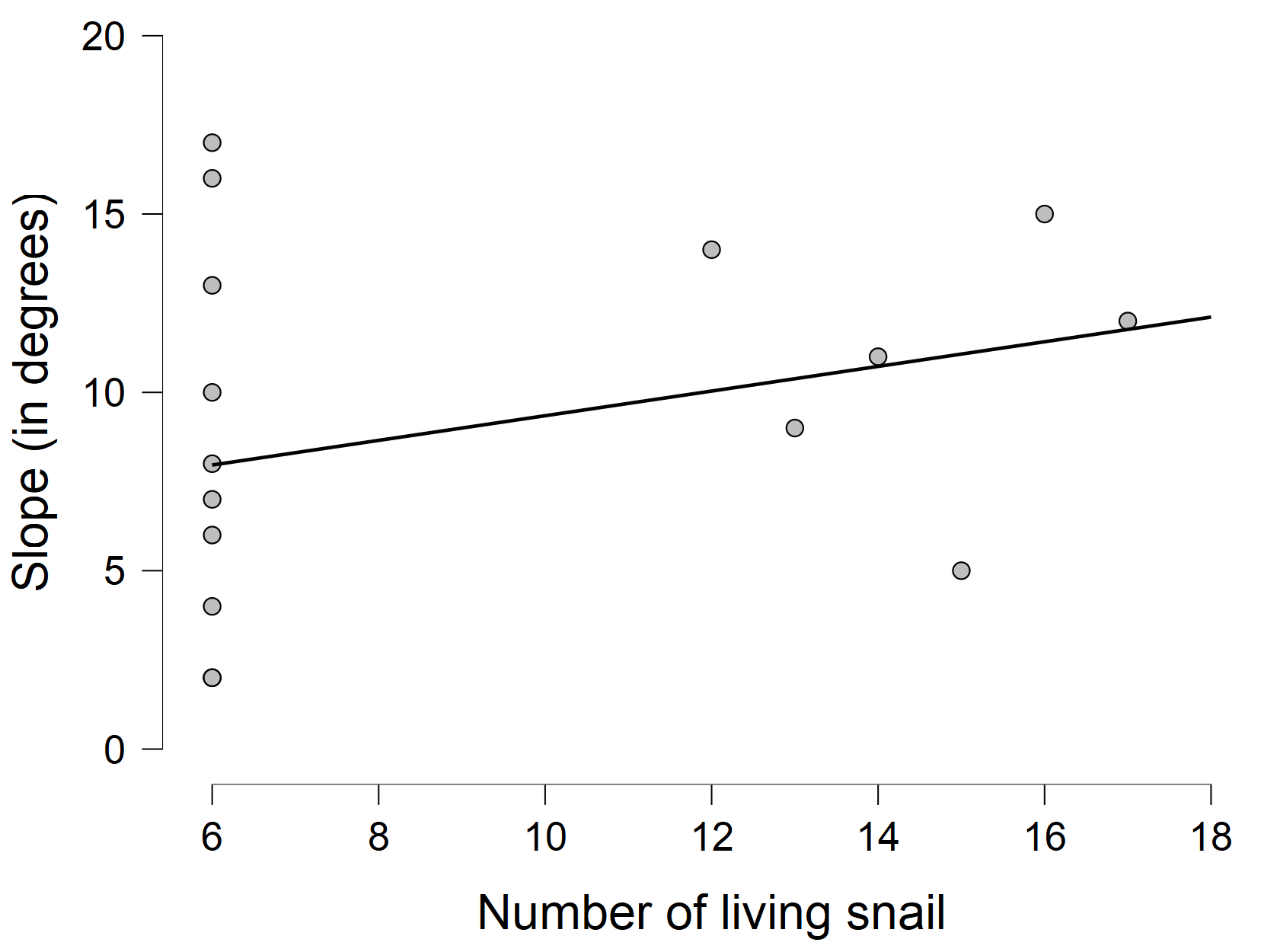

Supplement: Supplemental Information 3 — The dataset and the output of the analysis can be viewed by using JASP software version 0.12.2 (JASP Team, 2020). [file peerj-09-11886-s003.jasp › resources/1/_9_t1656675448.png]
