# Supplementary material for: Distribution and abundance of the land snail Pollicaria elephas (Gastropoda: Pupinidae) in limestone habitats in Perak, Malaysia
Source: PeerJ. 2021 Jul 28;9:e11886. doi: 10.7717/peerj.11886 (PMC8325424; doi:10.7717/peerj.11886)
Supplement: Supplemental Information 5 — The colour of the plots’ label represents the plot location on the centre, northern and southern parts of the limestone hill. Living Pollicaria elephas land snails were not found in any of the plots in the southern and central part of the limestone hills. In contrast, the snails were found in the six of the seven plots on the northern part of the same limestone hill (except A-P14 plot). [file peerj-09-11886-s005.docx]

**Additional File 5:** Principal components analysis (PCA) plots of the third axis with the first and the second axis, respectively, for habitat, topography and vegetation variables for all of the 17 plots on a limestone hill. The colour of the plots' label represents the plot location on the limestone hill. No individuals of *Pollicaria elephas* were found in plots at the southern and central part of the limestone hill, while at the northern part living snails were found in six of the seven plots (none were found in A-P14).


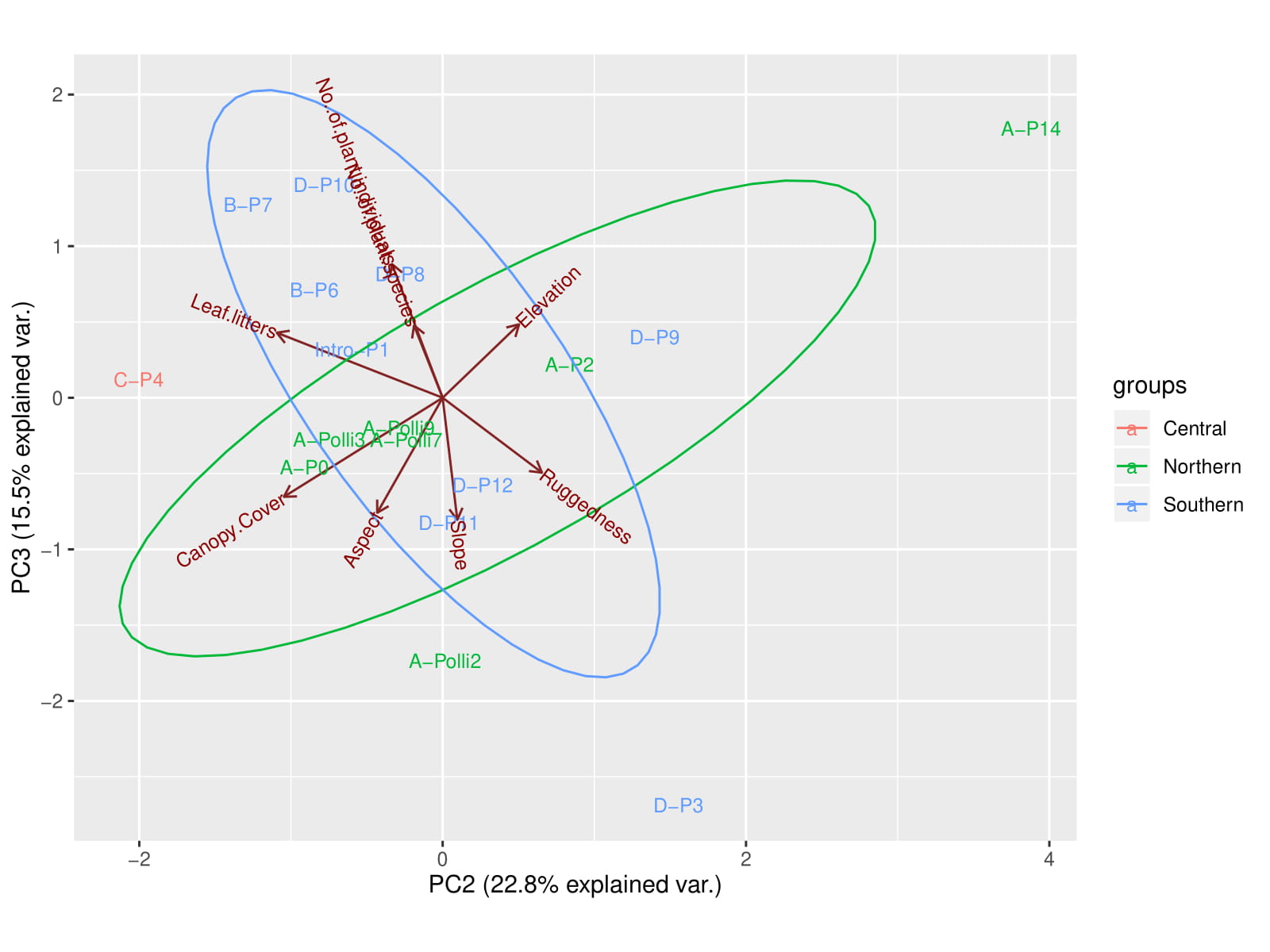
**Figure 1.** Principal components analysis (PCA) plots of the second axis with the third axis for habitat, topography and vegetation variables for all of the 17 plots on a limestone hill. The colour of the labels represents the plot location on the limestone hill and red vectors represent the habitat, topography, and vegetation variables.


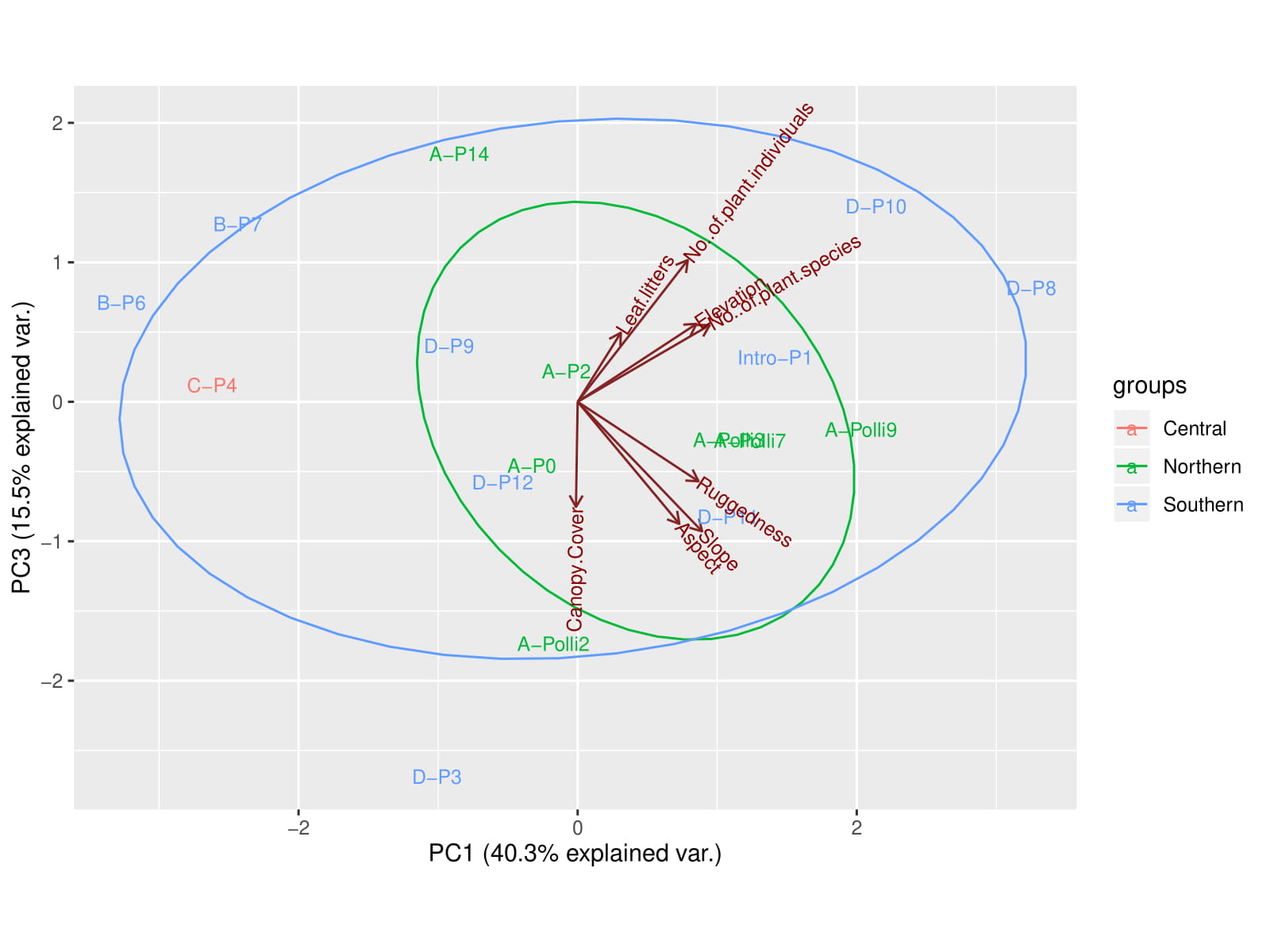
**Figure 2.** Principal components analysis (PCA) plots of the first axis with the third axis for habitat, topography and vegetation variables for all of the 17 plots on a limestone hill. The colour of the labels represents the plot location on the limestone hill and red vectors represent the habitat, topography, and vegetation variables.
