# Supplementary material for: Distribution and abundance of the land snail Pollicaria elephas (Gastropoda: Pupinidae) in limestone habitats in Perak, Malaysia
Source: PeerJ. 2021 Jul 28;9:e11886. doi: 10.7717/peerj.11886 (PMC8325424; doi:10.7717/peerj.11886)
Supplement: Supplemental Information 7 [file peerj-09-11886-s007.docx]

**Additional File 7. Correlation between the abundance of *Pollicaria elephas* for each plot with the abundance of the associated four vascular plant species**

**Table 1.** Correlation between the abundance of *Pollicaria elephas* for each plot with the abundance of the associated four vascular plant species.

| Family | Species | **Occurrence in the 17 plots** | Bayesian Kendall's Tau Correlations | BF₁₀ | Kendall's Tau Correlations | p-value |
| --- | --- | --- | --- | --- | --- | --- |
| Apocynaceae | *Kibatalia laurifolia* | 2 | **0.527*** | 17.738 | **0.527*** | 0.023 |
| Ebenaceae | *Diospyros toposia* var. *toposoides* | 8 | **0.519*** | 15.604 | **0.519*** | 0.015 |
| Euphorbiaceae | *Croton cascarilloides* | 2 | **0.499*** | 11.617 | **0.499*** | 0.030 |
| Euphorbiaceae | *Mallotus peltatus* | 3 | **0.547*** | 23.867 | **0.547*** | 0.016 |

*  BF₁₀ > 10, ** BF₁₀ > 30, *** BF₁₀ > 100; * p < 0.05, ** p < 0.01, *** p < 0.001, ᵃ The variance in *Macaranga tanarius* is equal to 0


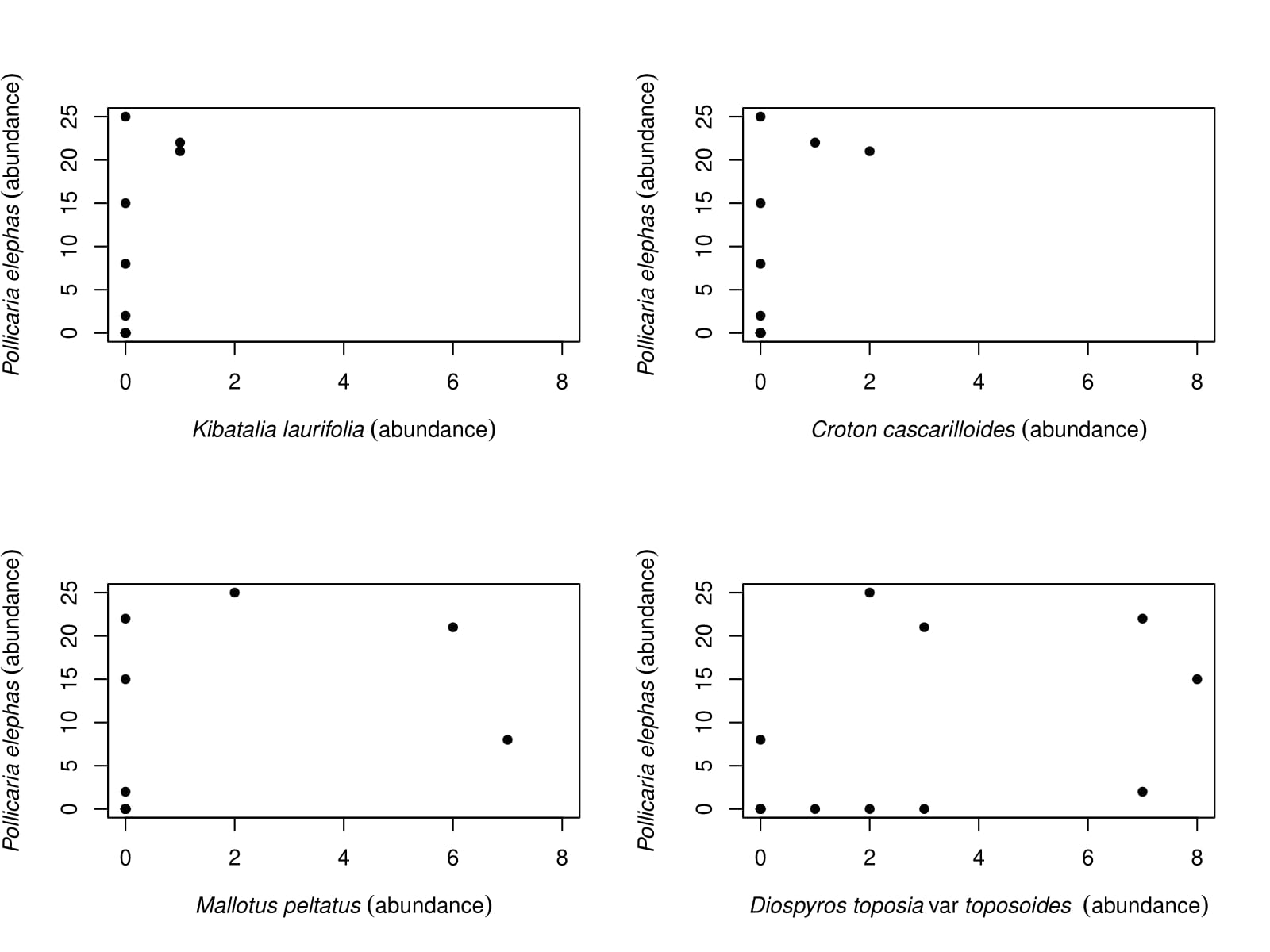


**Figure 1.** Correlation between the abundance of *Pollicaria elephas* for each plot with the abundance of the associated four vascular plant species.
