# Supplementary material for: Distribution and abundance of the land snail Pollicaria elephas (Gastropoda: Pupinidae) in limestone habitats in Perak, Malaysia
Source: PeerJ. 2021 Jul 28;9:e11886. doi: 10.7717/peerj.11886 (PMC8325424; doi:10.7717/peerj.11886)
Supplement: Supplemental Information 8 — A figure and raw data for mean soil temperature, air temperature and air humidity for every two hours of all the days in July 2018 in the plots. [file peerj-09-11886-s008.docx]

Additional File 8.


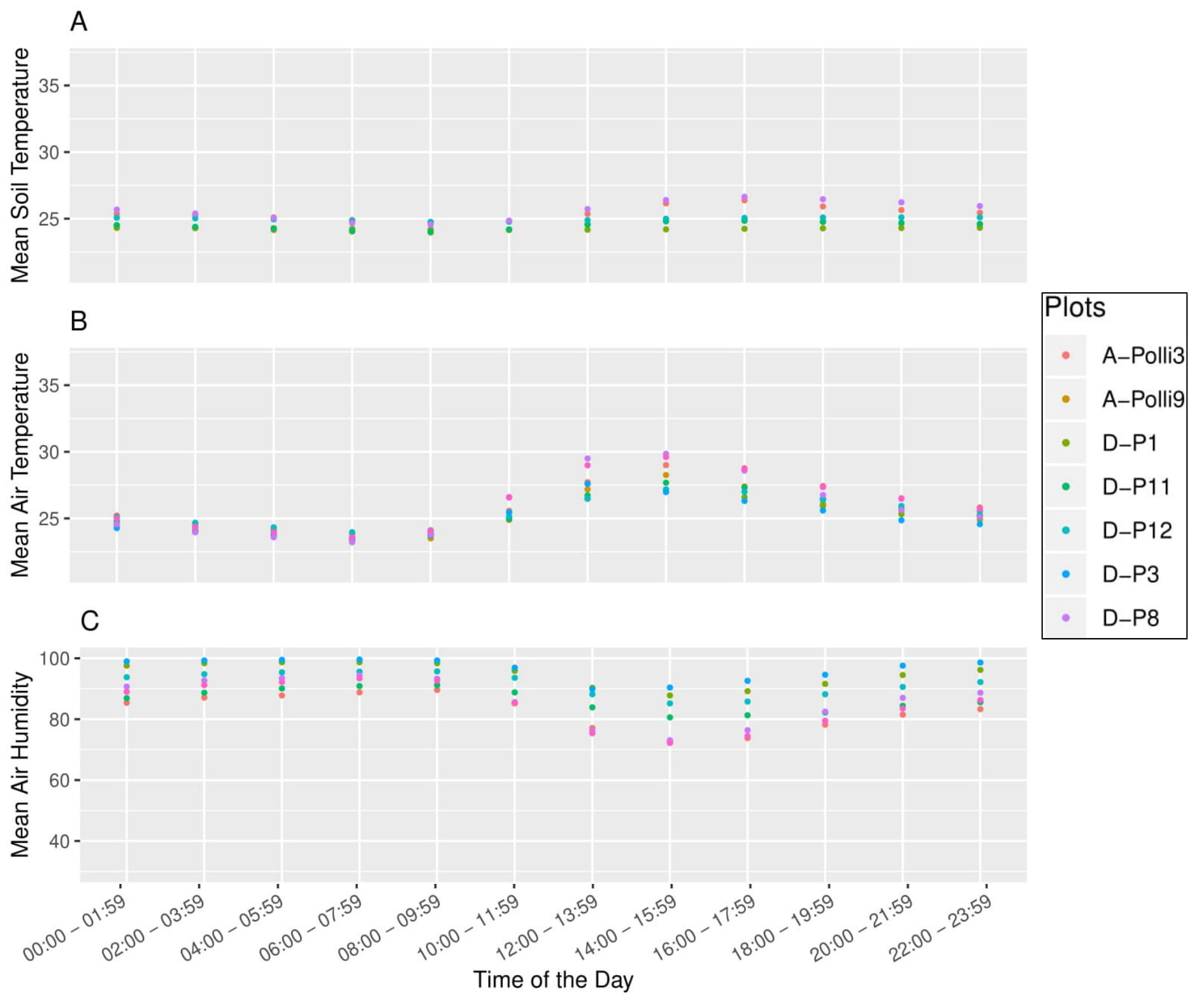


Figure 1. Mean soil temperature, air temperature and air humidity for every two hours of all the days in July 2018 in the plots. Plot A-Polli3 and A-Polli9 are the plots with living *Pollicaria elephas* land snails, while the other plots are without living *P. elephas* land snails. (A) Mean soil temperature. (B) Air temperature. (C) Air humidity.

Table 1. Mean soil temperature, air temperature and air humidity for every two hours of all the days in July 2018 in the plots.

| Plots | Time of the Day | Mean Air Temperature (°C) | Mean Air Humidity (%) | Mean Soil Temperature (°C) |
| --- | --- | --- | --- | --- |
| A-Polli3 | 00:00 - 01:59 | 25.21 | 85.4 | 25.36 |
| A-Polli3 | 02:00 - 03:59 | 24.6 | 87.1 | 25.27 |
| A-Polli3 | 04:00 - 05:59 | 24.22 | 87.8 | 25.09 |
| A-Polli3 | 06:00 - 07:59 | 23.82 | 88.8 | 24.9 |
| A-Polli3 | 08:00 - 09:59 | 23.95 | 89.6 | 24.75 |
| A-Polli3 | 10:00 - 11:59 | 25.57 | 85.2 | 24.81 |
| A-Polli3 | 12:00 - 13:59 | 27.72 | 77.1 | 25.36 |
| A-Polli3 | 14:00 - 15:59 | 29 | 72.5 | 26.15 |
| A-Polli3 | 16:00 - 17:59 | 28.76 | 73.8 | 26.38 |
| A-Polli3 | 18:00 - 19:59 | 27.43 | 78.2 | 25.92 |
| A-Polli3 | 20:00 - 21:59 | 26.51 | 81.5 | 25.65 |
| A-Polli3 | 22:00 - 23:59 | 25.82 | 83.3 | 25.45 |
| A-Polli9 | 00:00 - 01:59 | 24.61 | NA | 24.46 |
| A-Polli9 | 02:00 - 03:59 | 24.02 | NA | 24.29 |
| A-Polli9 | 04:00 - 05:59 | 23.73 | NA | 24.15 |
| A-Polli9 | 06:00 - 07:59 | 23.37 | NA | 24.04 |
| A-Polli9 | 08:00 - 09:59 | 23.5 | NA | 23.96 |
| A-Polli9 | 10:00 - 11:59 | 24.89 | NA | 24.16 |
| A-Polli9 | 12:00 - 13:59 | 27.18 | NA | 24.58 |
| A-Polli9 | 14:00 - 15:59 | 28.26 | NA | 24.9 |
| A-Polli9 | 16:00 - 17:59 | 27.4 | NA | 24.94 |
| A-Polli9 | 18:00 - 19:59 | 26.08 | NA | 24.77 |
| A-Polli9 | 20:00 - 21:59 | 25.55 | NA | 24.64 |
| A-Polli9 | 22:00 - 23:59 | 25.15 | NA | 24.56 |
| D-P1 | 00:00 - 01:59 | 24.49 | 97.6 | 24.31 |
| D-P1 | 02:00 - 03:59 | 24.12 | 98.4 | 24.31 |
| D-P1 | 04:00 - 05:59 | 23.84 | 98.7 | 24.29 |
| D-P1 | 06:00 - 07:59 | 23.53 | 98.7 | 24.24 |
| D-P1 | 08:00 - 09:59 | 23.85 | 98.4 | 24.19 |
| D-P1 | 10:00 - 11:59 | 25.01 | 95.9 | 24.16 |
| D-P1 | 12:00 - 13:59 | 26.48 | 90.3 | 24.17 |
| D-P1 | 14:00 - 15:59 | 27.02 | 87.8 | 24.2 |
| D-P1 | 16:00 - 17:59 | 26.59 | 89.2 | 24.24 |
| D-P1 | 18:00 - 19:59 | 25.92 | 91.6 | 24.28 |
| D-P1 | 20:00 - 21:59 | 25.32 | 94.5 | 24.3 |
| D-P1 | 22:00 - 23:59 | 24.92 | 96.2 | 24.32 |
| D-P11 | 00:00 - 01:59 | 24.81 | 86.9 | 24.52 |
| D-P11 | 02:00 - 03:59 | 24.37 | 88.7 | 24.39 |
| D-P11 | 04:00 - 05:59 | 24 | 90.1 | 24.25 |
| D-P11 | 06:00 - 07:59 | 23.57 | 90.9 | 24.08 |
| D-P11 | 08:00 - 09:59 | 23.71 | 91.2 | 24.01 |
| D-P11 | 10:00 - 11:59 | 24.97 | 88.8 | 24.2 |
| D-P11 | 12:00 - 13:59 | 26.73 | 83.9 | 24.55 |
| D-P11 | 14:00 - 15:59 | 27.68 | 80.6 | 24.8 |
| D-P11 | 16:00 - 17:59 | 27.32 | 81.3 | 24.84 |
| D-P11 | 18:00 - 19:59 | 26.47 | 82.2 | 24.76 |
| D-P11 | 20:00 - 21:59 | 25.72 | 84.4 | 24.69 |
| D-P11 | 22:00 - 23:59 | 25.23 | 85.6 | 24.61 |
| D-P12 | 00:00 - 01:59 | 25.11 | 93.8 | 25.07 |
| D-P12 | 02:00 - 03:59 | 24.67 | 94.8 | 25.03 |
| D-P12 | 04:00 - 05:59 | 24.33 | 95.4 | 24.95 |
| D-P12 | 06:00 - 07:59 | 23.96 | 95.6 | 24.84 |
| D-P12 | 08:00 - 09:59 | 24.11 | 95.7 | 24.75 |
| D-P12 | 10:00 - 11:59 | 25.14 | 93.6 | 24.77 |
| D-P12 | 12:00 - 13:59 | 26.49 | 88.2 | 24.89 |
| D-P12 | 14:00 - 15:59 | 27.18 | 85.2 | 24.99 |
| D-P12 | 16:00 - 17:59 | 27 | 85.8 | 25.06 |
| D-P12 | 18:00 - 19:59 | 26.4 | 88.2 | 25.09 |
| D-P12 | 20:00 - 21:59 | 25.93 | 90.6 | 25.1 |
| D-P12 | 22:00 - 23:59 | 25.55 | 92.2 | 25.1 |
| D-P3 | 00:00 - 01:59 | 24.28 | 99 | NA |
| D-P3 | 02:00 - 03:59 | 23.99 | 99.3 | NA |
| D-P3 | 04:00 - 05:59 | 23.72 | 99.5 | NA |
| D-P3 | 06:00 - 07:59 | 23.39 | 99.6 | NA |
| D-P3 | 08:00 - 09:59 | 23.74 | 99.3 | NA |
| D-P3 | 10:00 - 11:59 | 25.45 | 96.9 | NA |
| D-P3 | 12:00 - 13:59 | 27.59 | 89.9 | NA |
| D-P3 | 14:00 - 15:59 | 26.98 | 90.4 | NA |
| D-P3 | 16:00 - 17:59 | 26.31 | 92.6 | NA |
| D-P3 | 18:00 - 19:59 | 25.59 | 94.6 | NA |
| D-P3 | 20:00 - 21:59 | 24.86 | 97.6 | NA |
| D-P3 | 22:00 - 23:59 | 24.57 | 98.6 | NA |
| D-P8 | 00:00 - 01:59 | 24.56 | 90.7 | 25.68 |
| D-P8 | 02:00 - 03:59 | 23.96 | 92.7 | 25.39 |
| D-P8 | 04:00 - 05:59 | 23.59 | 93.5 | 25.04 |
| D-P8 | 06:00 - 07:59 | 23.21 | 94.4 | 24.67 |
| D-P8 | 08:00 - 09:59 | 23.78 | 93.3 | 24.56 |
| D-P8 | 10:00 - 11:59 | 26.57 | 85.6 | 24.85 |
| D-P8 | 12:00 - 13:59 | 29.5 | 76.1 | 25.72 |
| D-P8 | 14:00 - 15:59 | 29.85 | 73.1 | 26.4 |
| D-P8 | 16:00 - 17:59 | 28.59 | 76.4 | 26.64 |
| D-P8 | 18:00 - 19:59 | 26.76 | 82.5 | 26.46 |
| D-P8 | 20:00 - 21:59 | 25.63 | 87 | 26.23 |
| D-P8 | 22:00 - 23:59 | 25.1 | 88.7 | 25.95 |
| D-P9 | 00:00 - 01:59 | 25.01 | 89 | NA |
| D-P9 | 02:00 - 03:59 | 24.36 | 91.2 | NA |
| D-P9 | 04:00 - 05:59 | 23.95 | 92.2 | NA |
| D-P9 | 06:00 - 07:59 | 23.56 | 93.4 | NA |
| D-P9 | 08:00 - 09:59 | 24.07 | 92.7 | NA |
| D-P9 | 10:00 - 11:59 | 26.6 | 85.4 | NA |
| D-P9 | 12:00 - 13:59 | 28.99 | 75.4 | NA |
| D-P9 | 14:00 - 15:59 | 29.61 | 72.2 | NA |
| D-P9 | 16:00 - 17:59 | 28.7 | 74.5 | NA |
| D-P9 | 18:00 - 19:59 | 27.35 | 79.5 | NA |
| D-P9 | 20:00 - 21:59 | 26.48 | 83.5 | NA |
| D-P9 | 22:00 - 23:59 | 25.71 | 86.3 | NA |
